# One-pot synthesis of naphtho[1,2-*e*][1,3]oxazines in the presence of FNAOSiPAMP*/Cu^II^ as an almond shell based nanocatalyst

**DOI:** 10.1038/s41598-022-22712-0

**Published:** 2022-10-21

**Authors:** Mina Keihanfar, Bi Bi Fatemeh Mirjalili

**Affiliations:** Department of Chemistry, College of Science, Yazd University, Yazd, Iran

**Keywords:** Catalyst synthesis, Heterogeneous catalysis

## Abstract

In the present research work, a novel catalyst based on natural material, namely, Fe_3_O_4_@nano-almondshell@OSi(CH_2_)_3_/NHCH_2_pyridine/Cu^II^ abbreviated (FNAOSiPAMP/Cu^II^) was designed and prepared. The properties of the catalyst was identified by Fourier-transform infrared spectroscopy (FT-IR), Thermogravimetry ananlysis (TG), X-ray diffraction (XRD), Energy-dispersive X-ray spectroscopy (EDS), Field emission scanning electron microscopy (FESEM), Transmission electron microscopy (TEM), and Mapping. Furthermore, the evaluation of catalytic activity was done in the course of naphtho-1,3-oxazines synthesis. Solvent-free conditions, simplicity of operation, easy work-up and use of an eco-friendly catalyst are some of advantages of this protocol.

## Introduction

One-pot multicomponent reaction has several merits over the routine and step-by-step reaction. The advantages of one-pot multicomponent reactions are the rapid achievement of complexity and variety in the synthesis of organic materials through highly practical and time-saving approaches. Moreover, this synthetic tool allows chemists to meet the criteria of green chemistry, such as waste prevention, atom and step economy, saving of solvents and reagents, uncomplicated purification procedures, avoidance of hazardous materials, and energy efficiency^1–8^. Recently, chemists, by consideration of green chemistry law, choice eco-friendly synthetic methods such as solvent-free condition and using nanocatalyst for organic reactions^9–15^.

Cellulose, as a naturally abundant biopolymer and renewable resource containing OH groups is one of the most ideal coating layers for Fe_3_O_4_ NPs^16,17^. Almond shell is a natural and readily available source of cellulose. Heterogeneous catalyst based on magnetic nanoparticles have high dispersion in reaction mixture and simple removing by an external magnet advantages^18–21^. Cu^II^ as ecofriendly cation is a good Lewis acid and can active the carbonyl group for nucleophilic addition reactions^22^.

1,3-Oxazines^23–25^ have potential biological and pharmacological properties such as antibacterial^26^, analgesic^27^, antitumor^28^, antihypertensive^29^, anti-HIV^30^, antithrombotic^31^, antiulcer^32^, and anti-Parkinson’s disease^33^ activities. Recently, some protocols have been developed for the synthesis of benzo-fused 1,3-oxazines, such as, Mannich-type condensation of formaldehyde, β-naphthol, and amine^34^, acidic aza-acetalizations of aromatic aldehydes with 2-(*N*-substituted aminomethyl) phenols^35,36^, and electrooxidative cyclization of hydroxyamino compounds^37^. According to literature, the multicomponent reaction of formaldehyde, β-naphthol and amine 1° were done in the present of a catalyst such as ZrOCl_2_^38^, 1-benzyl-3-methyl imidazolium hydrogen sulfate [bnmim] [HSO_4_]^39^, PEG-400^40^, alum (KAl(SO_4_)_2_·12H_2_O)^41^, Thiamin hydrochloride (VB_1_)^42^ and Cl_3_CCOOH^43^.

Herein, we report an eco-friendly protocol for the synthesis of naphtho[1,2-*e*][1,3]oxazines in the presence of Fe_3_O_4_@nano-almondshell@OSi(CH_2_)_3_/NHCH_2_pyridine/Cu^II^, abbreviated, FNAOSiPAMP/Cu^II^, as a new natural-based green catalyst via the reaction of *β*-naphthol, primary amines and formaldehyde.

### General

Chemicals were purchased from Merck, Fluka, and Aldrich Chemical Companies. The electrical mortar-heater was prepared from Borna- Kherad Co., Iran, Yazd. ^1^H NMR (400 MHz) and ^13^C NMR (100 MHz) spectra were obtained by a Bruker (DRX-400, Avance), Fourier transform infrared (FT-IR) spectra recorded by ATR method on a Brucker (EQUINOX 55) spectrometer. Melting points were found on a B¨uchi B-540 instrument. The X-ray diffraction (XRD) spectra was obtained by a Philips Xpert MPD diffractometer equipped with a Cu Kα anode (*k* = 1.54 Å, radiation at 0 kV and 0 mA) in the 2θ range from 10° to 80°. Field Emission Scanning Electron Microscopy (FESEM) (MIRA 3 TESCAN) and TEM (CM120) apparatus was used for recording of FESEM and TEM images. A vibrating sample magnetometer (VSM, Meghnatis Daghigh Kavir Co. Kashan Kavir, Iran) was used for measurements of magnetic property of catalyst. Energy-dispersive X-ray spectrometer (EDS) and maps of catalyst were recorded by MIRA II Detector SAMX. Thermal gravimetric analysis (TGA) was done using SDT Q600 V20.9 Build 20 instrument. Inductively coupled plasma mass spectrometry (ICP-MS) was recorded by AGILENT 7500 apparatus.

## Experimental

### Synthesis of nano-almond shell

Firstly, 5 g of almond shell was well powdered and reacted with 80 ml of 17.5% NaOH solution under reflux conditions for 24 h. Then, almond shell was filtered and washed with distilled water. It was then bleached with 20 ml of sodium hypochlorite solution and 60 ml of distilled water under reflux conditions for 2 h. Subsequently, the almond shell was filtered and washed well with distilled water. The obtained almond shell powder was added to 80 ml of the 35% sulfuric acid aqueous solution and heated under reflux condition for 6–7 h. The resulting suspension was diluted with water and centrifuged many times to obtain the resulting nano-almond shell.

### Synthesis of Fe_3_O_4_@nano-almond shell

7 G (0.026 mol) of FeCl_3_·6H_2_O and 2.6 g (0.0130 mol) of FeCl_2_·4H_2_O were added to a mixture of 2 g nano-almondshell and 200 mL 0.05 M acetic acid and mixed for 4 h at 80 °C. Then, 12 mL of 25% NH_4_OH was added drop wise into the obtained mixture. After 0.5 h stirring, by using an external magnet, the obtained Fe_3_O_4_@nano-almondshell as a black solid (3 g) was separated, washed with water, dried at 80 °C and stored.

### Synthesis of Fe_3_O_4_@ nano-almondshell@OSi(CH_2_)_3_Cl

1G of Fe_3_O_4_@nano-almondshell and 3 ml 3-chloropropyl trimethoxysilane were dissolved in 10 ml chloroform. The reaction mixture was heated under reflux condition for 4 h. Then, the obtained precipitate was separated with external magnet, washed with dichloromethane and dried at room temperature.

### Synthesis of Fe_3_O_4_@nano-almondshell@OSi(CH_2_)_3_/NHCH_2_pyridine (FNAOSiPAMP)

0.5G of nano-Fe_3_O_4_@almondshell @OSi(CH_2_)_3_Cl and (1 mmol, 0.1 mL) 2-aminomethylpyridine were dissolved in 5 ml dimethylformamide and heated for 24 h at 80 °C. Then the obtained precipitate was filtered, washed with dichloromethane and dried at room temperature.

### Synthesis of Fe_3_O_4_@nano-almondshell@OSi(CH_2_)_***3***_/NHCH_***2***_pyridine/Cu^II^ (FNAOSiPAMP/Cu^II^)

0.5G of nano-Fe_3_O_4_@almondshell @OSi(CH_2_)_3_/NHCH_2_pyridine and (1 mmol, 0.17 g) of CuCl_2_ were dissolved in 5 ml methanol and stirred for 1 h. Then, the solid product was filtered, washed with methanol and dried.

### Synthesis of naphtho[1,2-e][1,3] oxazines

In an electrical mortar-heater vessel, amine 1° (1.0 mmol), formaldehyde 37% (2.0 mmol), *β*-naphthol (1.0 mmol) and FNAOSiPAMP/Cu^II^ (0.04 g) were charged and ground at room temperature for determined time. Finally, the obtained mixture was poured in hot ethanol (3 mL) and the catalyst was separated by using an external magnet. Then, cold water was added to residue and the obtained solid product was filtered, washed with water and dried at room temperature.

### Test of hot filtration and metal leaching

To study the leaching of (FNAOSiPAMP/Cu^II^), a hot filtration test was done. For hot filtration test, we have run a model reaction in the presence of catalyst. After ten minutes, the catalyst was removed from reaction mixture by an external magnet. The remained mixture was stirred for 15 min. The progress of reaction was not of observed and shown no leaching of catalyst in this protocol.

#### Spectral data for selected compounds

##### 3-Phenyl-2,4-dihydro-1H-naphtho[1,2-e][1,3]oxazine (Table 2, entry 1, 4a)

White solid, m.p. 45–47 °C; FT-IR (ATR) ῡ (cm^−1^): 3057, 1623, 1597, 1496, 1376, 1230, 941, 747; ^1^H NMR (Acetone-d_6_, 400 MHz)/δ ppm: 7.89 (d, 1H, ^3^* J* = 8.4 Hz, Ar–H), 7.85 (d, 1H, ^3^* J* = 8 Hz, Ar–H), 7.73 (d, 1H, ^3^* J* = 8.2 Hz, Ar–H), 7.53–7.57 (m, 1H, Ar–H), 7.38–7.42 (m, 1H, Ar–H), 7.23–7.28 (m, 4H, Ar–H), 7.04 (d, 1H, ^3^* J* = 8.2 Hz, Ar–H), 6.87–6.91 (m, 1H, Ar–H), 5.54 (s, 2H, O–CH_2_–N), 5.06 (s, 2H, –Ar–CH_2_–N). (See SI, Fig. S1-S3).

##### 3-(4-Bromophenyl)-2,4-dihydro-1H-naphtho[1,2-e][1,3]oxazine (Table 2, entry 2, 4b)

White solid, m.p. 118–119 °C; FT-IR (ATR) ῡ (cm^-1^): 3070, 2978,1622, 1590, 1487, 1371, 1223, 933, 808; ^1^H NMR (Acetone-d_6_, 400 MHz)/δ ppm: 7.84 (t, 2H, ^3^* J* = 8.2 Hz, Ar–H), 7.71 (d, 1H, ^3^* J* = 8.2 Hz, Ar–H), 7.52 (t, 1H, ^3^* J* = 8.4 Hz, Ar–H), 7.37–7.40 (m, 3H, Ar–H), 7.20 (d, 2H, ^3^*J* = 8.2 Hz, Ar–H), 7.01 (d, 1H, ^3^* J* = 8.2 Hz, Ar–H), 5.51 (s, 2H, O–CH_2_–N), 5.03 (s, 2H, –Ar–CH_2_–N); (See SI, Fig. S4, S5).

##### 3-(4-Chlorophenyl)-2,4-dihydro-1H-naphtho[1,2-e][1,3]oxazine (Table 2, entry 3, 4c)

White solid, m.p. 103–104 °C; FT-IR (ATR) ῡ (cm^−1^): 3307, 1622, 1593, 1491, 1369, 1224, 810; ^1^H NMR (Acetone-d_6_, 400 MHz)/δ ppm: 7.84 (t, 2H, ^3^*J* = 8.2 Hz, Ar–H), 7.71 (d, 1H, ^3^*J* = 8.2 Hz, Ar–H), 7.53 (t, 1H, ^3^*J* = 8.2 Hz, Ar–H), 7.38 (t, 1H, ^3^*J* = 8.0 Hz, Ar–H), 7.24–7.29 (m, 4H, Ar–H), 7.02 (d, 1H, ^3^*J* = 8.2 Hz, Ar–H), 5.51 (s, 2H, O–CH_2_–N), 5.03 (s, 2H, –Ar–CH_2_–N); (See SI, Fig. S6, S7).

##### 3-(4-Methoxyphenyl)-2,4-dihydro-1H-naphtho[1,2-e][1,3]oxazine (Table 2, entry 4, 4d)

Brown solid, m.p. 78–79 °C; FT-IR (ATR) ῡ (cm^−1^): 1623, 1596, 1508, 1467, 1229, 1156, 1032, 943, 805; ^1^H NMR (Acetone-d_6_, 400 MHz)/δ ppm: 7.80–7.82 (m, 2H, Ar–H), 7.70 (d, 1H, ^3^*J* = 8.2 Hz, Ar–H), 7.51 (m, 1H, Ar–H), 7.36 (m, 1H, Ar–H), 7.13 (d, 2H, ^3^*J* = 8.2 Hz, Ar–H), 7.00 (d, 1H, ^3^*J* = 8.2 Hz, Ar–H), 6.80 (d, 2H, ^3^* J* = 8.2 Hz, Ar–H), 5.42 (s, 2H, O–CH_2_–N), 4.93 (s, 2H, –Ar–CH_2_–N), 3.68 (s, 3H, O–CH_3_); (See SI, Fig. S8, S9).

##### 3-(4-Methyl phenyl)-2,4-dihydro-1H-naphtho[1,2-e][1,3]oxazine (Table 2, entry 5, 4e)

Cream solid, m.p. 84–85 °C; FT-IR (ATR) ῡ (cm^−1^): 1625, 1599, 1513, 1227, 936, 807; ^1^H NMR (Acetone-d_6_, 400 MHz)/δ ppm: 7.82 (t, 2H, ^3^*J* = 8.2 Hz, Ar–H), 7.69 (d, 1H, ^3^*J* = 8.2 Hz, Ar–H), 7.51 (t, 1H, ^3^*J* = 8.0 Hz, Ar–H), 7.36 (t, 1H, ^3^*J* = 8.0 Hz, Ar–H), 6.99–7.04 (m, 3H, Ar–H), 7.09–7.11 (m, 2H, Ar–H), 5.52 (s, 2H, O–CH_2_–N), 5.02 (s, 2H, –Ar–CH_2_–N), 2.24 (s, 3H, CH_3_–Ar); (See SI, Fig. S10, S11).

##### 3-Cyclohexyl-2,4-dihydro-1H-naphtho[1,2-e][1,3]oxazine (Table 2, entry 6, 4f.)

Brown solid, m.p. 254 °C; FT-IR (ATR) ῡ (cm^-1^): 2927, 2852, 1624, 1599, 1433, 1263, 1058, 862; ^1^H NMR (DMSO-d_6_, 500 MHz)/δ ppm: 7.66–7.81 (m, 3H, Ar–H), 7.48 (m, 1H, Ar–H), 7.35 (m, 1H, Ar–H), 6.98 (m, 1H, Ar–H), 4.99 (s, 2H, O–CH_2_–N), 4.33 (s, 2H, –Ar–CH_2_–N), 2.70 (m, 1H, CH–N), 1.08–1.86 (m, 10H, 5CH_2_); (See SI, Fig. S12, S13).

##### 3-Butyl-2,4-dihydro-1H-naphtho[1,2-e][1,3]oxazine (Table 2, entry 7, 4 g)

White solid, m.p. 170 °C; FT-IR (ATR) ῡ (cm^-1^): 2955, 2861, 1624, 1598, 1468, 1226, 1057; ^1^H NMR (DMSO-d_6_, 500 MHz)/δ ppm: 7.81 (m, 1H, Ar–H), 7.69 (m, 2H, Ar–H), 7.47 (m, 1H, Ar–H), 7.35 (m, 1H, Ar–H), 7.00 (m, 1H, Ar–H), 4.88 (s, 2H, O–CH_2_–N), 4.25 (s, 2H, –Ar–CH_2_–N), 2.69 (m, 2H, –CH_2_–N), 1.53 (m, 2H, CH_2_), 1.31 (m, 2H, CH_2_), 0.87 (m, 3H, CH_3_); ^13^C NMR (DMSO-d_6_, 100 MHz)/δ ppm: 13.80, 19.80, 29.64, 46.94, 50.84, 81.81, 112.08, 118.25, 121.31, 123.26, 126.45, 127.57, 128.32, 128.37, 131.54, 151.51 (See SI, Fig. S14-S16).

##### 3-Hexyl-2,4-dihydro-1H-naphtho[1,2-e][1,3]oxazine (Table 2, entry 8, 4 h)

Cream solid, m.p. 182–183 °C; FT-IR (ATR) ῡ (cm^−1^): 2927, 2855, 1625, 1598, 1468, 1226, 1058; ^1^H NMR (DMSO-d_6_, 500 MHz)/δ ppm: 7.80 (m, 1H, Ar–H), 7.68 (m, 2H, Ar–H), 7.47 (m, 1H, Ar–H), 7.34 (m, 1H, Ar–H), 7.00 (m, 1H, Ar–H), 4.87 (s, 2H, O–CH_2_–N), 4.25 (s, 2H, –Ar–CH_2_–N), 2.68 (m, 2H, –CH_2_–N), 1.53 (m, 2H, CH_2_), 1.25 (m, 6H, 3CH_2_), 0.84 (m, 3H, CH_3_); ^13^C NMR (DMSO-d_6_, 125 MHz)/δ ppm: 14.77, 22.98, 27.23, 28.38, 32.02, 47.87, 52.09, 82.71, 112.97, 119.14, 122.18, 124.13, 127.32, 128.46, 129.21, 129.28, 132.44, 152.42 (See SI, Fig. S17-S19).

##### 3-(5-Chloro-2-methyl phenyl)-2,4-dihydro-1H-naphtho[1,2-e][1,3]oxazine (Table 2, entry 9, 4i)

White solid, m.p. 151–152 °C; FT-IR (ATR) ῡ (cm^−1^): 2918, 1623, 1591, 1470, 1266, 1058; ^1^H NMR (DMSO-d_6_, 400 MHz)/δ ppm: 7.87 (m, 1H, Ar–H), 7.79 (m, 2H, Ar–H), 7.52 (m, 1H, Ar–H), 7.41(m, 1H, Ar–H), 7.12 (m, 3H, Ar–H), 7.07 (m, 1H, Ar–H), 5.28 (s, 2H, O–CH_2_–N), 4.76 (s, 2H, –Ar–CH_2_–N), 2.5 (m, 2H, –CH_2_–N), 2.40 (m, 2H, CH_2_); (See SI, Fig. S20-S21).

##### 3-(4-Ethyl phenyl)-2,4-dihydro-1H-naphtho[1,2-e][1,3]oxazine (Table 2, entry 10, 4j)

Brown solid, m.p. 48–49 °C; FT-IR (ATR) ῡ (cm^−1^): 2959, 1597, 1511, 1436, 1224, 1060; ^1^H NMR (Acetone-d_6_, 400 MHz)/δ ppm: 7.80–7.85 (m, 2H, Ar–H), 7.69–7.71 (m, 1H, Ar–H), 7.50–7.55 (m, 1H, Ar–H), 7.35–7.40 (m, 1H, Ar–H), 6.99–7.14 (m, 5H, Ar–H), 5.49 (s, 2H, O–CH_2_–N), 5.00 (s, 2H, –Ar–CH_2_–N), 2.49–2.54 (m, 2H, –CH_2_–CH_3_), 1.15 (t, 3H, ^3^*J* = 7 Hz, –CH_2_–CH_3_); (See SI, Fig. S22-S23).

##### 3-(2-Chlorobenzyl)-2,4-dihydro-1H-naphtho[1,2-e][1,3]oxazine (Table 2, entry 11, 4 k)

Brown solid, m.p. 72–73 °C; FT-IR (ATR) ῡ (cm^−1^): 1621, 1596, 1429, 1227, 1057; ^1^H NMR (DMSO-d_6_, 500 MHz)/δ ppm: 7.07–7.82 (m, 10H, Ar–H), 4.95 (s, 2H, O–CH_2_–N), 4.27 (s, 2H, –Ar–CH_2_–N), 4.00 (s, 2H, –Ar–CH_2_–N); ^13^C NMR (DMSO-d_6_, 100 MHz)/δ ppm: 46.82, 52.56, 81.94, 111.73, 118.33, 121.26, 123.42, 126.46, 127.23, 127.85, 128.39, 128.50, 128.90, 129.35, 130.61, 131.54, 133.23, 135.81, 151.41 (See SI, Fig. S24-S26).

##### 3-Benzyl-2,4-dihydro-1H-naphtho[1,2-e][1,3]oxazine (Table 2, entry 12, 4l)

White solid, m.p. 143–144 °C; FT-IR (ATR) ῡ (cm^-1^): 2943, 1623, 1596, 1462, 1219, 1057, 738; ^1^H NMR (DMSO-d_6_, 500 MHz)/δ ppm: 7.05–7.71 (m, 11H, Ar–H), 4.89 (s, 2H, O–CH_2_–N), 4.22 (s, 2H, –Ar–CH_2_–N), 3.89 (s, 2H, –Ar–CH_2_–N); ^13^C NMR (DMSO-d_6_, 100 MHz)/δ ppm: 46.55, 55.17, 81.55, 111.67, 118.32, 121.15, 123.37, 126.57, 127.19, 127.76, 128.34, 128.39, 128.49, 128.59, 131.53, 138.33, 151.41 (See SI, Fig. S28-S30).

##### 3-(2-Bromobenzyl)-2,4-dihydro-1H-naphtho[1,2-e][1,3]oxazine (Table 2, entry 13, 4m)

Brown solid, m.p. 120–122 °C; FT-IR (ATR) ῡ (cm^−1^): 2880,1623, 1597, 1507, 1476, 1229,1070; ^1^H NMR (DMSO-d_6_, 400 MHz)/δ ppm: 7.91 (t, 2H, ^3^*J* = 9.2 Hz, Ar–H), 7.72 (d, 1H, ^3^*J* = 9.2 Hz, Ar–H), 7.59(t, 1H, ^3^* J* = 8.4 Hz, Ar–H), 7.42 (m, 3H, Ar–H), 7.24(d, 2H, ^3^*J* = 9.2 Hz, Ar–H), 7.03(d, 1H, ^3^*J* = 9.2 Hz, Ar–H), 5.36 (s, 2H, O–CH_2_–N), 4.86 (s, 2H, –Ar–CH_2_–N); (See SI, Fig. S31, S32).

## Results and discussion

**Figure 1:**
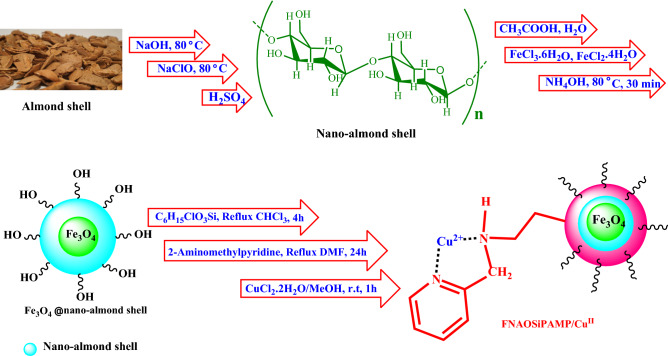
Graphical representation of FNAOSiPAMP/Cu^II^ synthesis.

In this research, we have prepared FNAOSiPAMP/Cu^II^ catalyst via a simple procedure that shown in Fig. 1. After identify the properties of catalyst by FT-IR, FESEM, TEM, EDS, ICP, MAPPING, VSM, TGA and XRD, we have introduced an efficient and eco-friendly protocol for the synthesis of naphtho[1,2-*e*][1,3]oxazine derivatives in the presence of FNAOSiPAMP/Cu^II^ catalyst.

## Characterization of FNAOSiPAMP/Cu^II^

### FT-IR analysis

**Figure 2:**
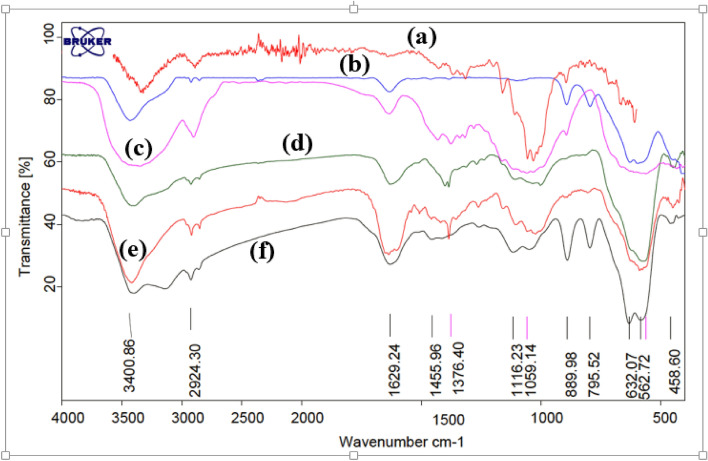
FT-IR spectra of (**a**) almondshell, (**b**) Fe_3_O_4_ , (**c**) Fe_3_O_4_@nano-almondshell, (**d**) Fe_3_O_4_@nano-almondshell@OSi(CH_2_)_3_Cl, (**e**) FNAOSiPAMP, (**f**) FNAOSiPAMP/Cu^II^.

Figure 2 shows the FT-IR spectra of nano-almond shell (a), Fe_3_O_4_ (b), Fe_3_O_4_@nano-almondshell (c), Fe_3_O_4_@nano-almondshell@OSi(CH_2_)_3_Cl (d), FNAOSiPAMP (e) and FNAOSiPAMP/Cu^II^ (f). The bands at 3400 cm^−1^ in all spectra (a–f) and 2924 cm^−1^ in (a, c, d, e and f) spectra, are attributed to O–H and C–H stretching vibration. The band at 1629 cm^−1^ in all spectra (a–f) shows bending vibration of O–H band. The band at 1455 cm^−1^ in (a, c, d, e and f) spectra shows CH_2_ bending vibration. The band at 1116 cm^−1^ in (d, e, f) spectra and 1056 cm^−1^ in (a, c, d, e and f) spectra shows stretching vibration od Si–O and C–O bands, respectively. The broad band nearly 600 cm^−1^ in (b–f) spectra shows Fe/O group. The broad band at 3200 cm^-1^ in (f) spectrum is attributed to N–H stretching vibration which is bonded to Cu^II^.

**Figure 3:**
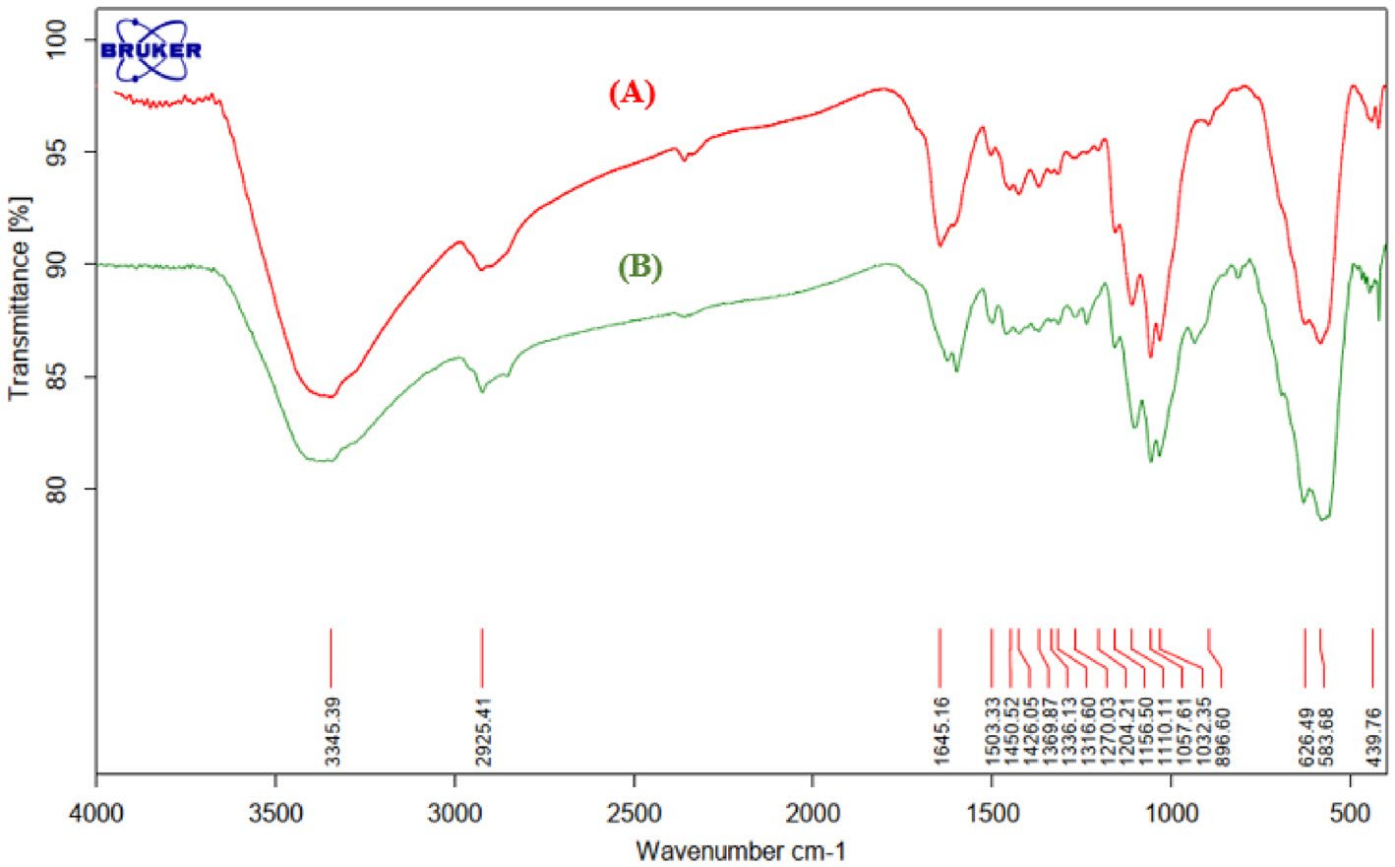
FT-IR spectrum of (**A**) fresh FNAOSiPAMP/Cu^II^, (**B**) used FNAOSiPAMP/Cu^II^.

A comparison between FTIR of fresh and used catalyst shows no any leaching of catalyst under reaction condition (Fig. 3.).

### XRD analysis

**Figure 4:**
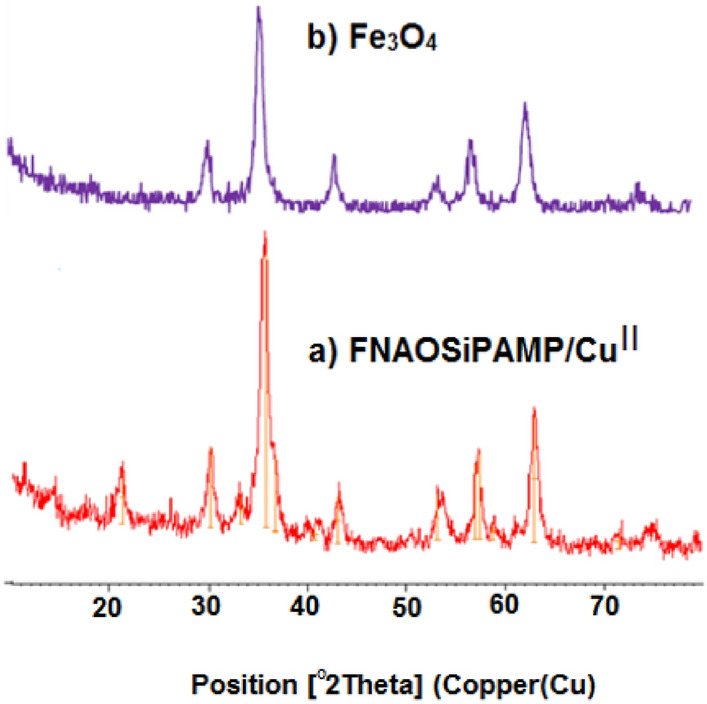
XRD patterns of, (**a**) FNAOSiPAMP/Cu^II^, (**b**) Fe_3_O_4_.

Figure 4 shows the XRD patterns of Fe_3_O_4_ and FNAOSiPAMP/Cu^II^, in 10°–80°. In XRD spectrum of FNAOSiPAMP/Cu^II^, in addition of Fe_3_O_4_ signals (2θ = 30°, 35°, 43°, 53°, 57°and 63°), 2θ = 21° shown the existence of almondshell.

### FESEM and TEM imaging

**Figure 5:**
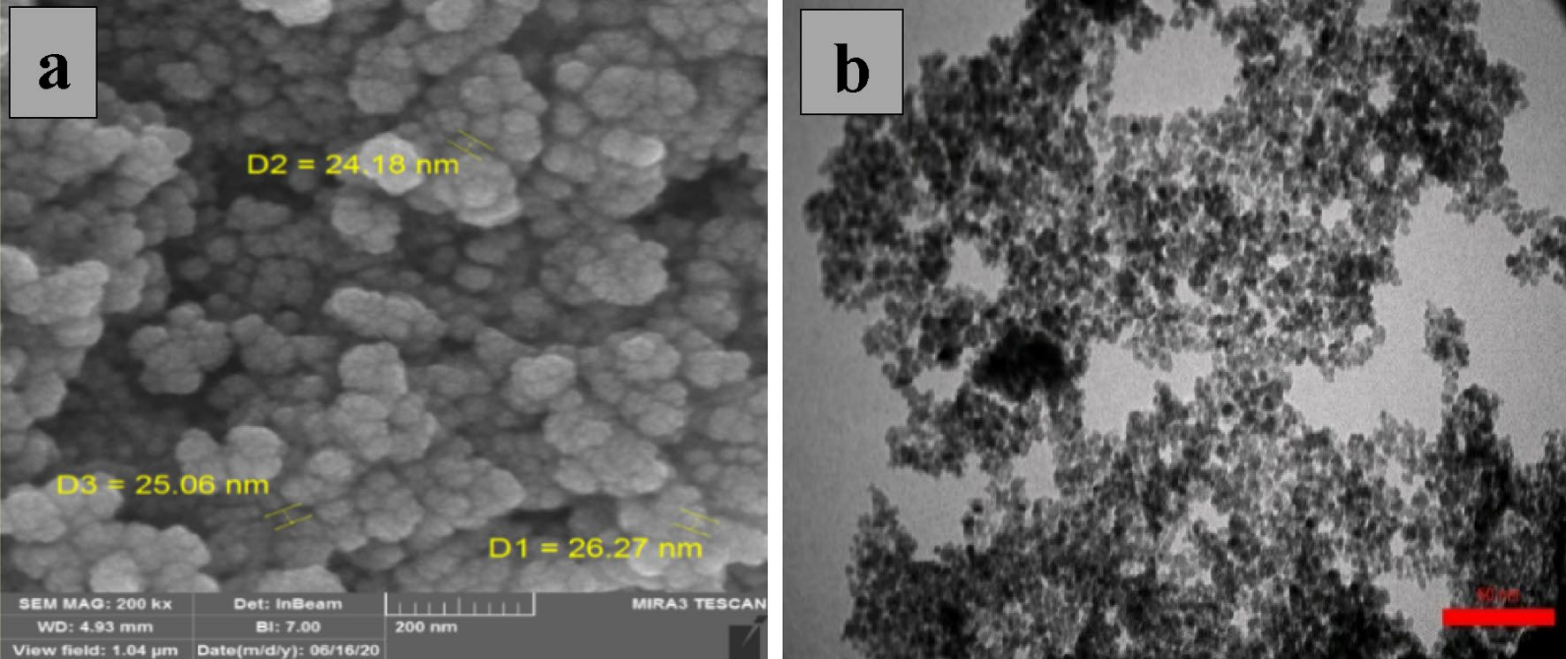
(**a**) FESEM and (**b**) TEM image of FNAOSiPAMP/Cu^II^.

Figure 5 represents the result of field emission scanning electron microscopy (FESEM) and transmission electron microscopy (TEM) of FNAOSiPAMP/Cu^II^ to investigate its particle size and surface morphology. This images indicates that FNAOSiPAMP/Cu^II^ nanoparticles have average size below 30 nm. The presence of spherical particles with nano dimensions in the catalyst increases the contact surface between the catalyst and the raw materials and increases the speed of the reaction.

### VSM analysis

**Figure 6:**
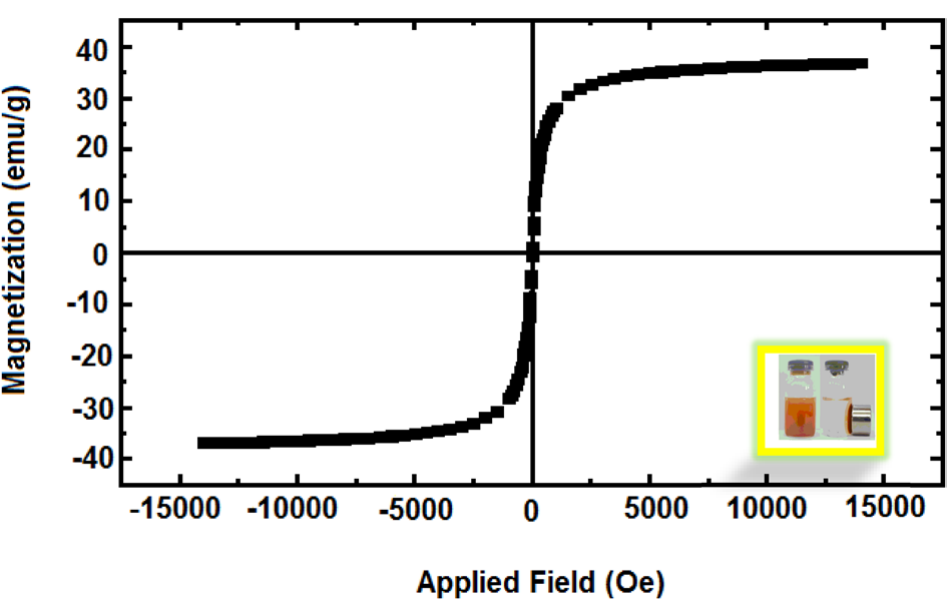
Magnetization loops of FNAOSiPAMP/Cu^II^.

Vibrating sample magnetometer (VSM) was used for the study magnetic property of catalyst at 300 °K (Fig. 6). This experiment approves the superparamagnetic property of catalyst which can be efficiently separated from reaction medium with an external magnet.

### EDX and ICP analysis

**Figure 7:**
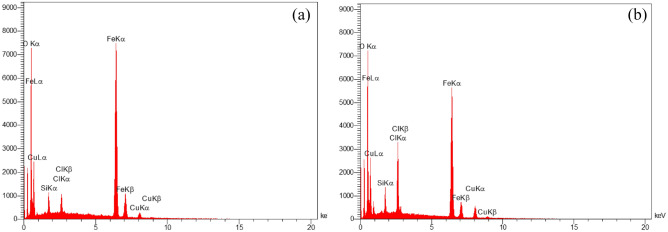
EDX patterns of (**a**) fresh FNAOSiPAMP/Cu^II^, (**b**) used FNAOSiPAMP/Cu^II^.

Energy-dispersive X-ray spectroscopy EDS (EDX) analysis was applied for the identification of elements in FNAOSiPAMP/Cu^II^ (Fig. 7). The EDX data confirmed the existence of C, N, O, Fe, elements in the catalyst. For approve no any leaching of catalyst under reaction condition, the EDX of fresh and used catalyst were shown in Fig. 7.

To finding the weight percentages of Cu and Fe in catalyst structure we have used ICP-MS. According to obtained data, the weight percentages of Cu and Fe are 3% and 15%, respectively.

### TG analysis

**Figure 8:**
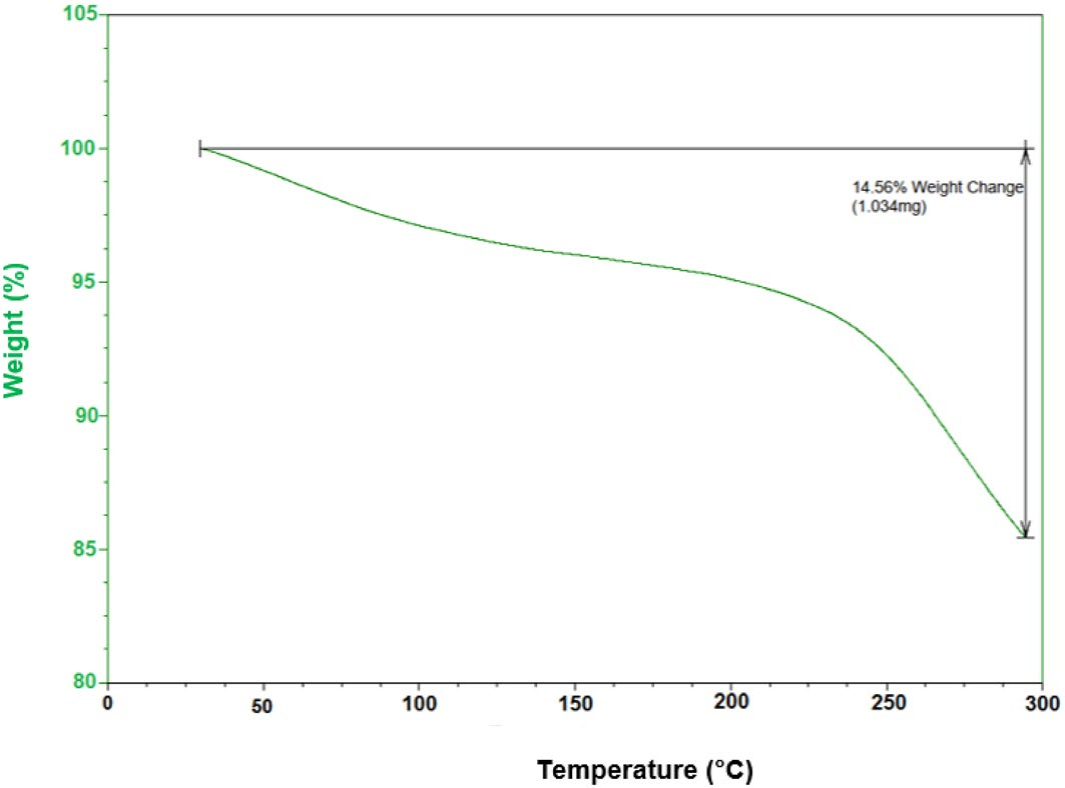
Thermal gravimetric analysis pattern of FNAOSiPAMP/Cu^II^.

TG analysis was performed to study thermal stability of the FNAOSiPAMP/Cu^II^ in 30°–300° (Fig. 8). The first endothermic weight loss (3–4%, at 50°–100°) was attributed to removal of catalyst humidity. Subsequently, the decomposition of almondshell, caused the second weight loss (15%) at 200°–300°.

### Mapping analysis

**Figure 9:**
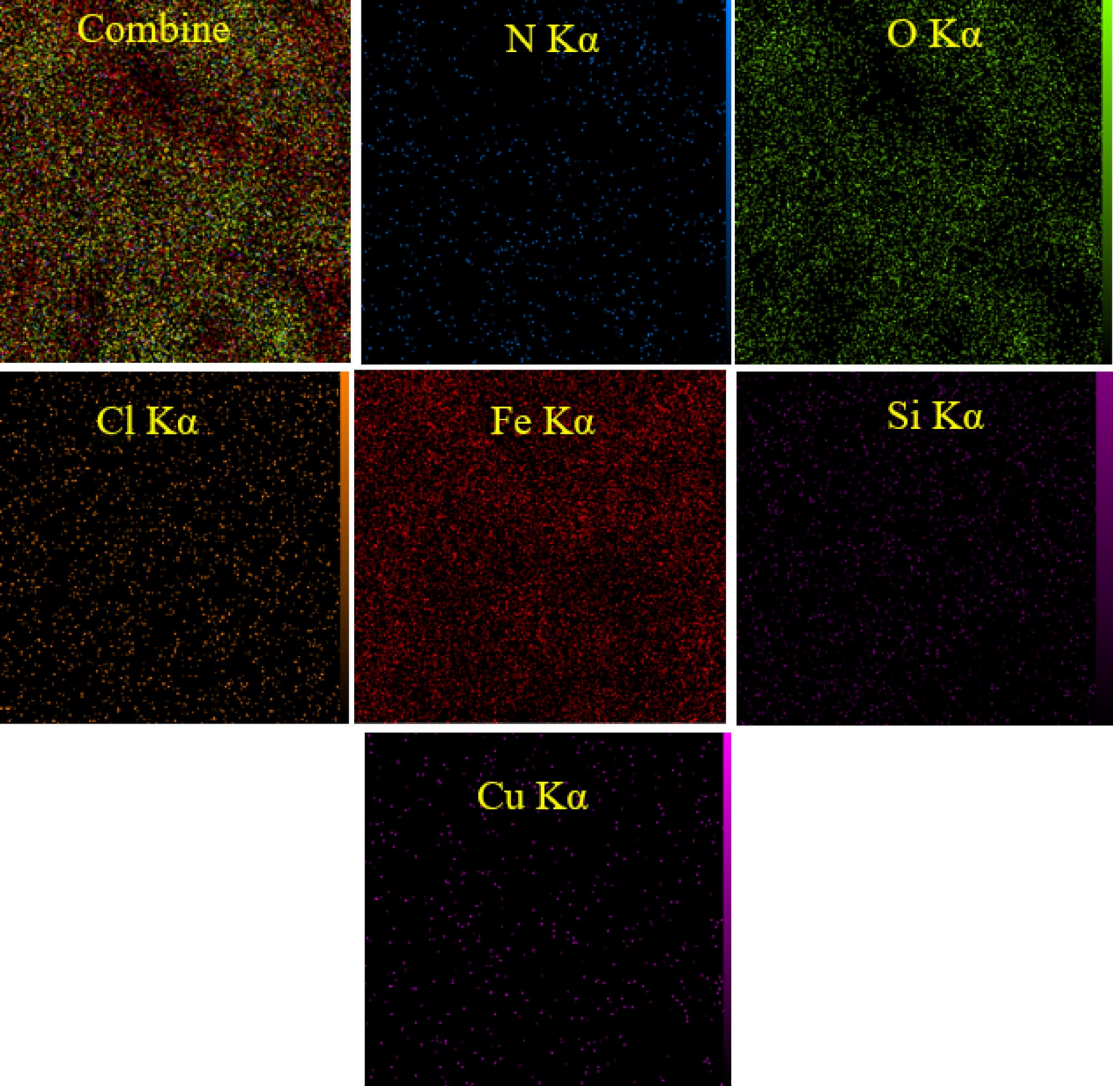
Elemental mapping images of FNAOSiPAMP/Cu^II^.

The elemental mapping of FNAOSiPAMP/Cu^II^ was shown in Fig. 9 which confirmed homogeneous distribution of N, O, C, Fe, Si and Cu in catalyst.

### Study of FNAOSiPAMP/Cu^II^ catalytic activity in the synthesis of naphtho[1,2-e][1,3]oxazines

Following the successful characterization, the catalytic activity of FNAOSiPAMP/Cu^II^ was investigated by synthesizing biological active naphtho[1,2-*e*][1,3]oxazines. To optimize the reaction conditions, the one-pot multicomponent reaction of formaldehyde, aniline and β-naphthol was studied in various conditions (Table 1). In conclusion, the best condition of this reaction was obtained using 0.04 g of FNAOSiPAMP/Cu^II^, solvent-free and room temperature conditions (Table 1, entry 10).

**Table 1:** The reaction of formaldehyde, β-naphthol and aniline in the presence of FNAOSiPAMP/Cu^II^ under various conditions.


Entry	Solvent	Conditions (C)	Catalyst (g)	Time (min)	Yield^a^ (%)
1	H_2_O	r.t	0.04	1440	80
2	–	40	0.04	60	65
3	–	Mixer mill	0.04	75	–
4	H_2_O	60	0.04	90	43
5	EtOH	r.t	0.04	300	24
6	EtOH	60	0.04	60	21
7	EtOH	75	0.04	120	27
8	–	r.t^b^	0.03	45	15
9	–	r.t^b^	0.02	50	–
10	–	r.t^b^	0.04	25	99

The molar ratio of formaldehyde (2 mmol), β-naphthol (1 mmol) and aniline (1 mmol) is equal to 2:1:1.

^a^Isolated yields.

^b^Electrical mortar-heater.

According to above modified condition, we have synthesized various derivatives of naphtho[1,2-*e*][1,3]oxazine using different amines, β-naphthol and formaldehyde with good to excellent yields in short reaction times (Table 2).

**Table 2:** Synthesis of naphtho[1,2-*e*][1,3]oxazines derivatives 4a-m by using various aniline derivatives, formaldehyde and β-naphthol in the presence of FNAOSiPAMP/Cu^II^.


Entry	Ar (R)	Product	Time (min)	Yield^a^ (%)	TON (TOF) (min^−1^)	M.P. (°C)
1	C_6_H_5_-	4a	25	99	52.6 (2.10638)	45–47
2	4-Br-C_6_H_4_ –	4b	10	91	48.4 (4.84042)	118–119
3	4-Cl-C_6_H_4_–	4c	25	87	46.2 (1.85106)	103–104
4	4-OMe-C_6_H_4_–	4d	30	75	39.8 (1.32978)	78–79
5	4-Me- C_6_H_4_–	4e	35	89	47.3 (1.35258)	84–85
6	(Cyclohexyl–)	4f	15	90	47.8 (3.19148)	254
7	(n-Butyl–)	4g	20	85	45.2 (2.26063)	170
8	(n-Hexyl–)	4h	25	95	50.5 (2.02127)	182–183
9	5-Cl-2-Me-C_6_H_3_–	4i	40	92	48.9 (1.22340)	151–152
10	4-Et–C_6_H_4_–	4j	30	91	48.4 (1.61347)	48–49
11	(2-Cl–C_6_H_4_–CH_2_–)	4k	60	90	47.8 (0.79782)	72–73
12	(C_6_H_5_–CH_2_–)	4l	50	95	50.5 (1.01063)	143–144
13	2-Br-C_6_H_4_–	4m	30	95	50.5 (1.68439)	120–122

The amount ratio of amine 1°, formaldehyde and β-naphthol is equal to 1:2:1.

^a^Isolated yields.

According to ICP data, the amount of Cu^II^ in catalyst is 3%. We have used 0.04 g of catalyst for 1 mmol of substrate (benzaldehyde). Thus 0.04 g of catalyst is containing of 1.2 × 10^–3^ g of Cu^II^ and equal to 1.88 × 10^–5^ mol of Cu^II^. Thus, TON and TOF of catalyst are equal to 52.6 and 2.10638 min^−1^, respectively.

**Figure 10:**
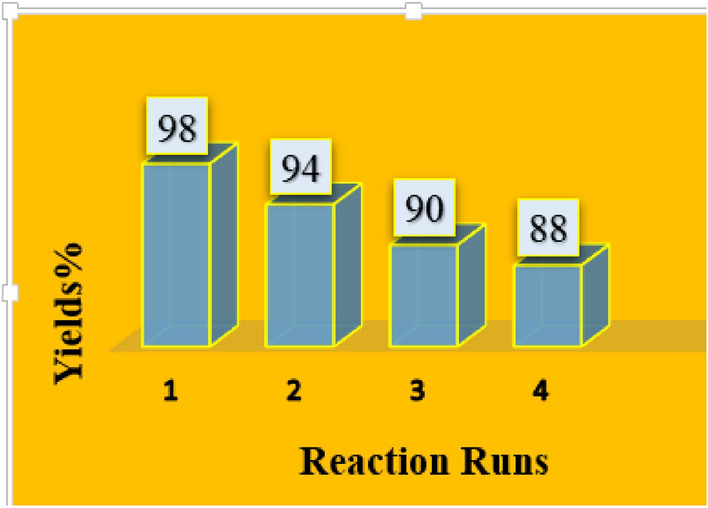
Catalyst recycling experiments.

The reusability of the magnetic nano-catalyst was examined in model reaction for four times. The result showed no considerable decrease of catalytic activity (Fig. 10). Meanwhile, no decomposition of catalyst in the reaction medium was confirmed by study of the reused catalyst FT-IR spectrum.

**Figure 11:**
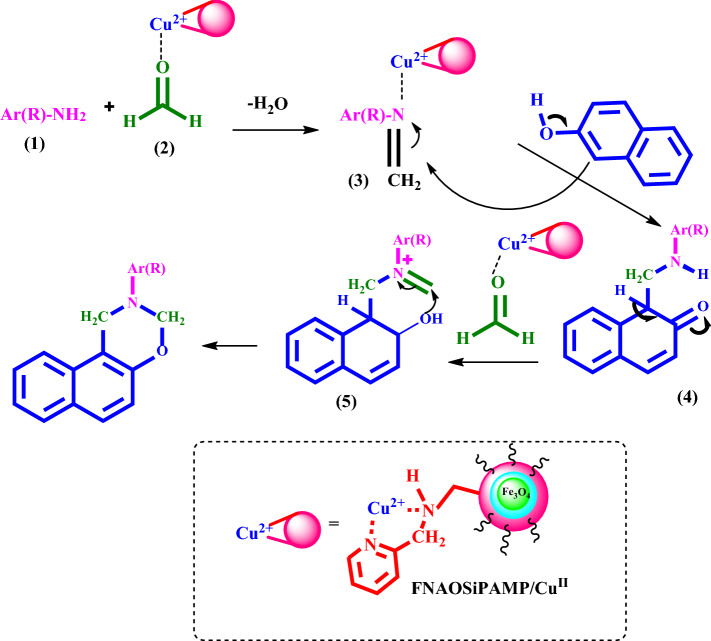
Proposed mechanism for the synthesis of naphtho[1,2-*e*][1,3]oxazines.

Figure 11 shows the our proposed mechanism for synthesis of naphtho[1,2-*e*][1,3]oxazines in the presence of FNAOSiPAMP/Cu^II^. Cu^II^ activates the carbonyl group in formaldehyde and then Mannich type condensation of the amine (1) and the formaldehyde (2) gives imine (3). In the next step, the *β*-naphthol attackes to the imine (3) to form intermediate (4) which condenses with the second molecule of formaldehyde to form intermediate (5). Then, by an intramolecular cyclization, the naphtho[1,2-*e*][1,3]oxazine is synthesized.

## Conclusions

In summary, this work introduces that FNAOSiPAMP/Cu^II^ as an effective novel catalyst promote a versatile, simple and environmentally benign protocol for the synthesis of naphtho[1,2-*e*][1,3]oxazines. This novel ecofriendly procedure for synthesis of naphtho[1,2-*e*][1,3]oxazine has many advantages such as simplicity, easy workup, reusability of catalyst, high yields and solvent-free reaction conditions of which turn it into a suitable alternative for the naphtho[1,2-*e*][1,3]oxazines synthesis.

## Supplementary Information

Supplementary Information.

## Data Availability

All data generated or analyzed during this study are included in this published article and its supplementary information files.

## References

[1] Cioc RC, Ruijter E, Orru RV (2014). Multicomponent reactions: advanced tools for sustainable organic synthesis. Green Chem..

[2] Kakuchi R (2014). Multicomponent reactions in polymer synthesis. Angew. Chem. Int. Ed..

[3] Garbarino S, Ravelli D, Protti S, Basso A (2016). Photoinduced multicomponent reactions. Angew. Chem. Int. Ed..

[4] Levi L, Müller TJ (2016). Multicomponent syntheses of functional chromophores. Chem. Soc. Rev..

[5] Ahmadi T, Ziarani GM, Gholamzadeh P, Mollabagher H (2017). Recent advances in asymmetric multicomponent reactions (AMCRs). Tetrahedron Asymmetry.

[6] Allochio Filho JF, Lemos BC, deSouza AS, Pinheiro S, Greco SJ (2017). Multicomponent Mannich reactions: General aspects, methodologies and applications. Tetrahedron.

[7] Ibarra IA, Islas-Jácome A, González-Zamora E (2018). Synthesis of polyheterocycles via multicomponent reactions. Org. Biomol. Chem..

[8] Zarganes-Tzitzikas T, Chandgude AL, Dömling A (2015). Multicomponent reactions, union of MCRs and beyond. Chem. Rec..

[9] Tobiszewski M, Mechlińska A, Namieśnik J (2010). Green analytical chemistry—Theory and practice. Chem. Soc. Rev..

[10] Dunn PJ (2012). The importance of green chemistry in process research and development. Chem. Soc. Rev..

[11] Simon M-O, Li C-J (2012). Green chemistry oriented organic synthesis in water. Chem. Soc. Rev..

[12] Anastas P, Eghbali N (2010). Green chemistry: Principles and practice. Chem. Soc. Rev..

[13] Maleki A, Niksefat M, Rahimi J, Taheri-Ledari R (2019). Multicomponent synthesis of pyrano [2, 3-d] pyrimidine derivatives via a direct one-pot strategy executed by novel designed copperated Fe_3_O_4_@ polyvinyl alcohol magnetic nanoparticles. Mater. Today Chem..

[14] Maleki A (2018). Green oxidation protocol: Selective conversions of alcohols and alkenes to aldehydes, ketones and epoxides by using a new multiwall carbon nanotube-based hybrid nanocatalyst via ultrasound irradiation. Ultrason Sonochem..

[15] Hajizadeh Z, Radinekiyan F, Eivazzadeh-Keihan R, Maleki A (2020). Development of novel and green NiFe_2_O_4_/geopolymer nanocatalyst based on bentonite for synthesis of imidazole heterocycles by ultrasonic irradiations. Sci. Rep..

[16] Maleki A, Kamalzare M (2014). Fe_3_O_4_@ cellulose composite nanocatalyst: preparation, characterization and application in the synthesis of benzodiazepines. Catal. Commun..

[17] Edjlali L, Khanamiri RH, Abolhasani J (2015). Fe_3_O_4_ nano-particles supported on cellulose as an efficient catalyst for the synthesis of pyrimido [4, 5-b] quinolines in water. Monatsh. Chem..

[18] Zolfigol MA, Moosavi-Zare AR, Moosavi P, Khakyzadeh V, Zare A (2013). Nano-ferrous ferric oxide (nano-Fe_3_O_4_): Powerful, reusable, and stable catalyst for N-Boc protection of amines. C. R. Chim..

[19] Maleki A, Hajizadeh Z, Salehi P (2019). Mesoporous halloysite nanotubes modified by CuFe_2_O_4_ spinel ferrite nanoparticles and study of its application as a novel and efficient heterogeneous catalyst in the synthesis of pyrazolopyridine derivatives. Sci. Rep..

[20] Maleki A, Hassanzadeh-Afruzi F, Varzi Z, Esmaeili MS (2020). Magnetic dextrin nanobiomaterial: An organic-inorganic hybrid catalyst for the synthesis of biologically active polyhydroquinoline derivatives by asymmetric Hantzsch reaction. Mater. Sci. Eng. C.

[21] Maleki A, Firouzi-Haji R (2018). L-Proline functionalized magnetic nanoparticles: A novel magnetically reusable nanocatalyst for one-pot synthesis of 2, 4, 6-triarylpyridines. Sci. Rep..

[22] Maleki A, Panahzadeh M, Eivazzadeh-keihan R (2019). Agar: A natural and environmentally-friendly support composed of copper oxide nanoparticles for the green synthesis of 1, 2, 3–triazoles. Green Chem. Lett. Rev..

[23] Safajoo N, Mirjalili BBF, Bamoniri A (2019). Fe_3_O_4_@ nano-cellulose/Cu (II): A bio-based and magnetically recoverable nano-catalyst for the synthesis of 4 H-pyrimido [2, 1-b] benzothiazole derivatives. RSC Adv..

[24] Bansal, R. K. *Heterocyclic Chemistry* (New Age International Publishers, 2014).

[25] Acheson, R. M. *An Introduction to The Chemistry of Heterocyclic Compounds* (Wiley India, 1960).

[26] Mathew BP, Kumar A, Sharma S, Shukla PK, Nath M (2010). An eco-friendly synthesis and antimicrobial activities of dihydro-2H-benzo- and naphtho-1,3-oxazine derivatives. Eur. J. Med. Chem..

[27] Kurz T (2005). Synthesis of novel pyrido [2, 3-e][1, 3] oxazines. Tetrahedron.

[28] Chylińska J, Urbański T, Mordarski M (1963). Dihydro-1, 3-oxazine derivatives and their antitumor activity. J. Med. Chem..

[29] Kajino M, Shibouta Y, Nishikawa K, Meguro K (1991). Synthesis and biological activities of new 2-substituted 1, 4-benzoxazine derivatives. Chem. Pharm. Bull..

[30] Cocuzza AJ (2001). Synthesis and evaluation of efavirenz (SustivaTM) analogues as HIV-1 reverse transcriptase inhibitors: replacement of the cyclopropylacetylene side chain. Bioorg. Med. Chem. Lett..

[31] Buckman BO (1998). Design, synthesis, and biological activity of novel purine and bicyclic pyrimidine factor Xa inhibitors. Bioorg. Med. Chem. Lett.

[32] Katsura Y, Nishino S, Takasugi H (1991). Studies on antiulcer drugs—I: Synthesis and antiuler activities of imidazo [1, 2-a] pyridinyl-2-oxobenzoxazolidines-3-oxo-2H-1, 4-benzoxazines and related compounds. Chem. Pharm. Bull..

[33] Joyce JN (2003). Neuroprotective effects of the novel D3/D2 receptor agonist and antiparkinson agent, S32504, in vitro against 1-methyl-4-phenylpyridinium (MPP+) and in vivo against 1-methyl-4-phenyl-1, 2, 3, 6-tetrahydropyridine (MPTP): a comparison to ropinirole. Exp. Neurol..

[34] Burke W, Murdock K, Ec G (1954). Condensation of hydroxyaromatic compounds with formaldehyde and primary aromatic amines. J. Am. Chem. Soc..

[35] Tang Z (2012). Synthesis and fungicidal activity of novel 2, 3-disubstituted-1, 3-benzoxazines. Molecules.

[36] Dong Y (2004). Highly regioselective N-alkylation of nonracemic Betti base: a novel one-pot synthesis of chiral N-methyl-N-alkyl Betti bases. Tetrahedron Asymmetry.

[37] Okimoto M (2012). Electrooxidative cyclization of hydroxyamino compounds possessing a benzyl group. Synthesis.

[38] Kategaonkar AH (2010). An efficient synthesis of 3, 4-dihydro-3-substituted-2H-naphtho [2, 1-e][1, 3] oxazine derivatives catalyzed by zirconyl (IV) chloride and evaluation of its biological activities. Bull. Korean Chem. Soc..

[39] Kategaonkar AH, Sonar SS, Shelke KF, Shingate BB, Shingare MS (2010). Ionic liquid catalyzed multicomponent synthesis of 3, 4-dihydro-3-substituted-2H-naphtho [2, 1-e][1, 3] oxazine derivatives. Org. Commun..

[40] Shinde PV, Kategaonkar AH, Shingate BB, Shingare MS (2011). Polyethylene glycol (PEG) mediated expeditious synthetic route to 1, 3-oxazine derivatives. Chin. Chem. Lett..

[41] Sadaphal SA, Sonar SS, Shingate BB, Shingare MS (2010). Water mediated synthesis of various [1, 3] oxazine compounds using alum as a catalyst. Green Chem. Lett. Rev..

[42] Dhakane VD, Gholap SS, Deshmukh UP, Chavan HV, Bandgar BP (2014). An efficient and green method for the synthesis of [1, 3] oxazine derivatives catalyzed by thiamine hydrochloride (VB_1_) in water. C. R. Chim..

[43] Borah R, Dutta AK, Sarma P, Dutta C, Sarma B (2014). Synthesis of anti-2, 3-dihydro-1, 2, 3-trisubstituted-1 H-naphth [1, 2-e][1, 3] oxazine derivatives via multicomponent approach. RSC Adv..

