# Supplementary material for: One-pot synthesis of naphtho[1,2-e][1,3]oxazines in the presence of FNAOSiPAMP*/CuII as an almond shell based nanocatalyst
Source: Sci Rep. 2022 Oct 21;12:17713. doi: 10.1038/s41598-022-22712-0 (PMC9587238; doi:10.1038/s41598-022-22712-0)
Supplement: Supplementary file 1 — Supplementary Information. [file 41598_2022_22712_MOESM1_ESM.docx]

**One-pot synthesis of naphtho[1,2-*e*][1,3]oxazines in the presence of FNAOSiPAMP*/Cu^II^ as an almond shell based nanocatalyst.**

***** Fe_3_O_4_@nano-almondshell@OSi(CH_2_)_3_/NHCH_2_pyridine

Mina Keihanfar and Bi Bi Fatemeh Mirjalili*

Department of Chemistry, College of Science, Yazd University, Yazd, Iran

*Email:fmirjalili@yazd.ac.ir Telephone: +983531232672, Fax: +98 3538210644*

**Synthesis of naphtho[1,2-*e*][1,3] oxazines**

In an electrical mortar-heater vessel, amine 1° (1.0 mmol), formaldehyde 37% (2.0 mmol), β-naphthol (1.0 mmol) and FNAOSiPAMP/Cu^II^ (0.04 g) were charged and ground at room temperature for determined time. Finally the obtained mixture was poured in hot ethanol (3 mL) and the catalyst was separated by using an external magnet. Then, cold water was added to residue and the obtained solid product was filtered, washed with water and dried at room temperature.

**2-Phenyl-2,3-dihydro-1*H*-naphtho[1,2-*e*][1,3]oxazine ( Table 2, Entry 1, 4a)**

White solid, m.p. 45-47 ^°^C; FT-IR (ATR) ῡ (cm^-1^): 3057, 1623, 1597, 1496, 1376, 1230, 941, 747(**Fig. S1**); ^1^H NMR (Acetone-d_6_, 400 MHz) /δ ppm: 7.89 (d, 1H, ^3^*J*= 8.4 Hz, Ar-H), 7.85 (d, 1H, ^3^*J*= 8 Hz, Ar-H), 7.73 (d, 1H, ^3^*J*= 9.2 Hz, Ar-H), 7.53-7.57 (m, 1H, Ar-H), 7.38-7.42 (m, 1H, Ar-H), 7.23-7.28 (m, 4H, Ar-H), 7.04 (d, 1H, ^3^*J*= 8.8 Hz, Ar-H), 6.87-6.91 (m, 1H, Ar-H), 5.54 (s, 2H, O-CH_2_-N), 5.06 (s, 2H, -Ar-CH_2_-N). (**Fig. S2-S3**).


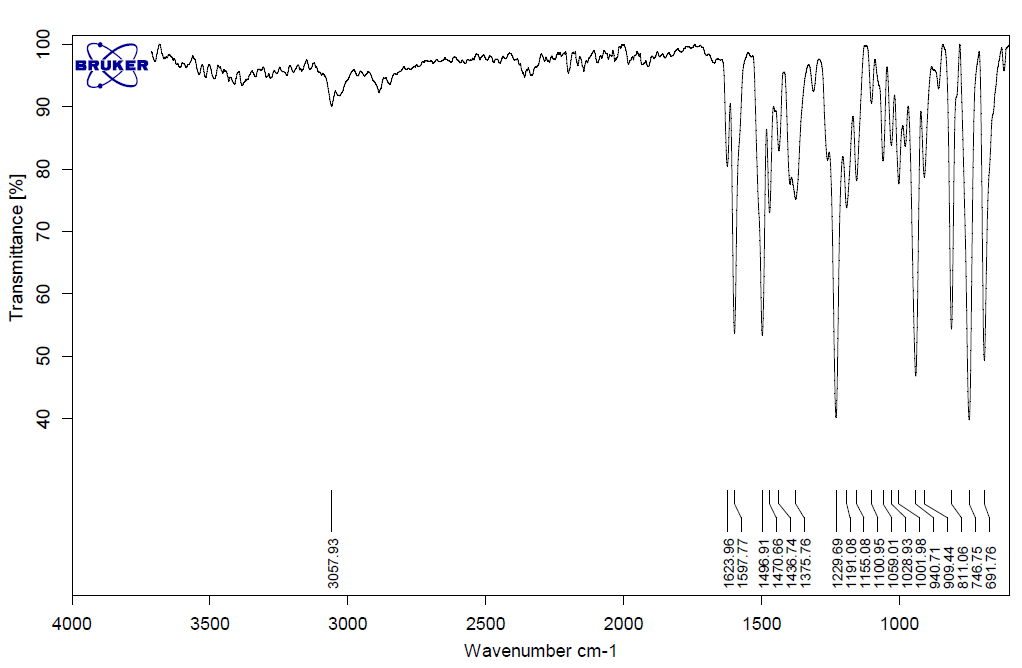


**Fig. S1.** The FT-IR spectrum of product (**4a**)

**Figure S2**. ^1^H NMR spectrum of compound (**4a**)

**Figure S3**. ^1^H NMR spectrum of compound (**4a**)

**2-(4-Bromophenyl)-2,3-dihydro-1*H*-naphtho[1,2-*e*][1,3]oxazine(Table 2, Entry 2, 4b)**

White solid, m.p. 118-119 ^°^C; FT-IR (ATR) ῡ (cm^-1^): 3070, 2978,1622, 1590, 1487, 1371, 1223,933, 808 (**Fig. S4**); ^1^H NMR (Acetone-d_6_, 400 MHz) /δ ppm: 7.84 (t, 2H, ^3^*J*= 9.2 Hz, Ar-H), 7.71 (d, 1H, ^3^*J*= 9.2 Hz, Ar-H), 7.52 (t, 1H, ^3^*J*= 8.4 Hz, Ar-H), 7.37-7.40 (m, 3H, Ar-H), 7.20 (d, 2H, ^3^*J*= 9.2 Hz, Ar-H), 7.01 (d, 1H, ^3^*J*= 9.2 Hz, Ar-H), 5.51 (s, 2H, O-CH_2_-N), 5.03(s, 2H, -Ar-CH_2_-N); (**Fig. S5**).


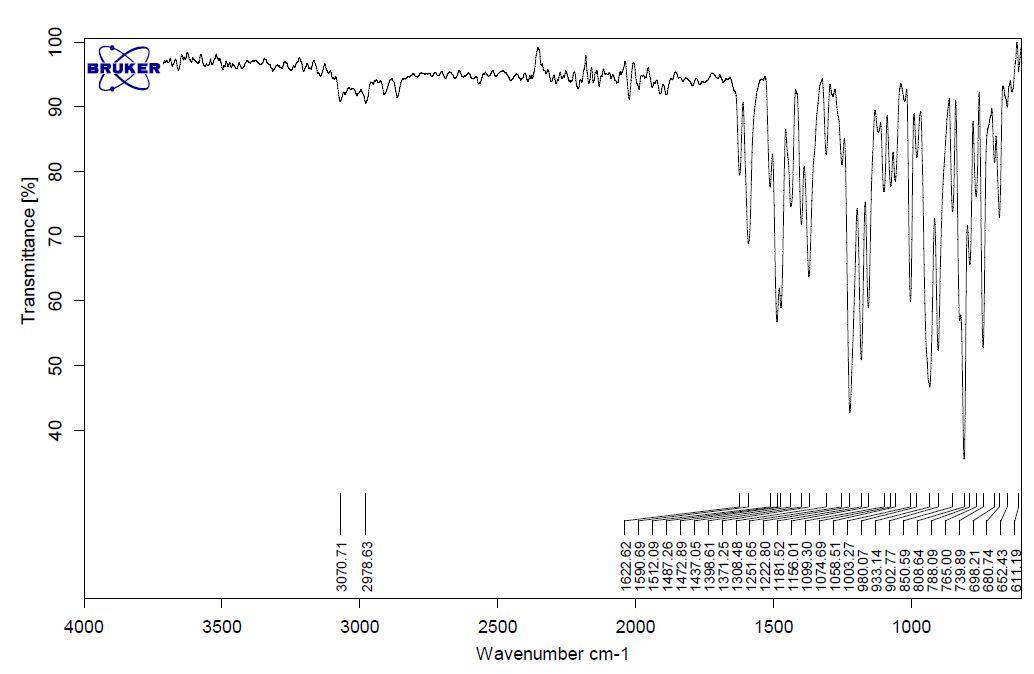


**Fig. S4.** The FT-IR spectrum of product (**4b**)

**Figure S5**. ^1^H NMR spectrum of compound (**4b**)

**2-(4-Chlorophenyl)-2,3-dihydro-1*H*-naphtho[1,2-*e*][1,3]oxazine (Table 2, Entry 3, 4c)**

White solid, m.p. 103-104 ^°^C; FT-IR (ATR) ῡ (cm^-1^): 3307, 1622, 1593, 1491, 1369, 1224, 810 (**Fig. S6**); ^1^H NMR (Acetone-d_6_, 400 MHz) /δ ppm: 7.84 (t, 2H, ^3^*J*= 9.2 Hz, Ar-H), 7.71 (d, 1H, ^3^*J*= 8.8 Hz, Ar-H), 7.53 (t, 1H, ^3^*J*= 7.2 Hz, Ar-H), 7.38 (t, 1H, ^3^*J*= 7.6 Hz, Ar-H), 7.24-7.29 (m, 4H, Ar-H), 7.02 (d, 1H, ^3^*J*= 8.8 Hz, Ar-H), 5.51 (s, 2H, O-CH_2_-N), 5.03(s, 2H, -Ar-CH_2_-N); (**Fig.S7**).


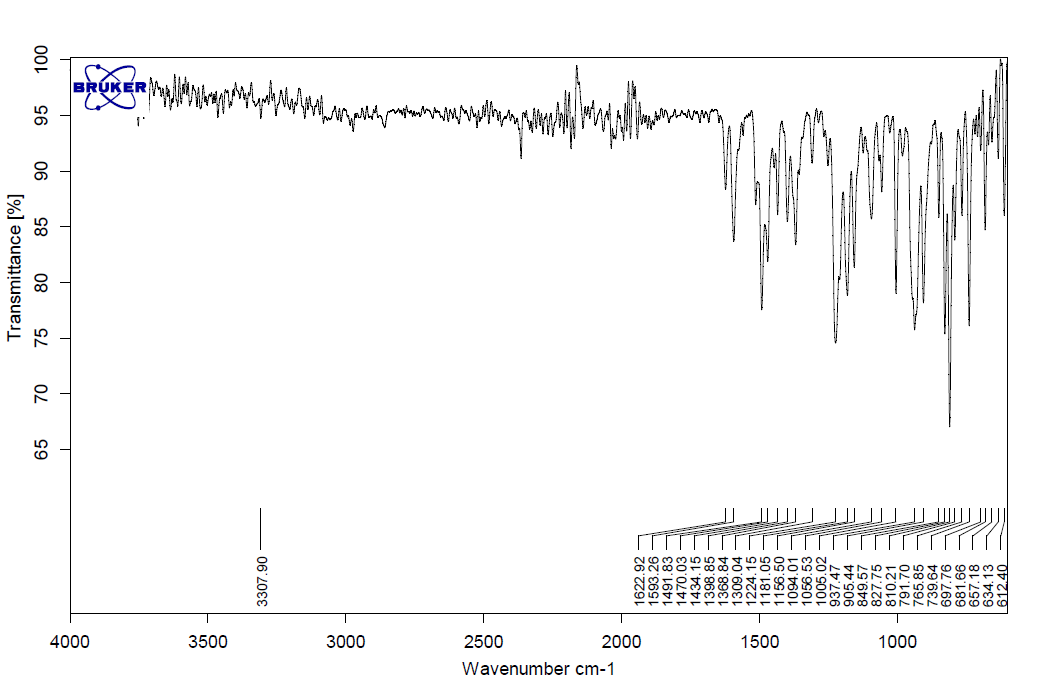


**Fig. S6.** The FT-IR spectrum of product (**4c**)


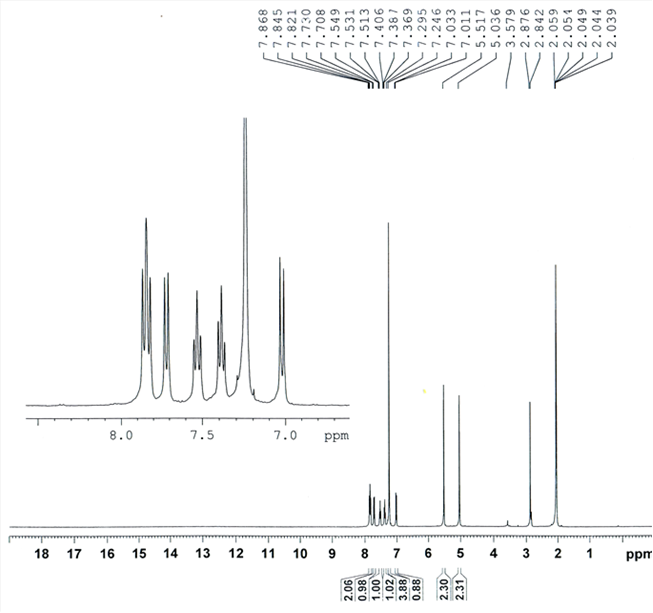


**Figure S7**. ^1^H NMR spectrum of compound (**4c**)

**2-(4-Methoxyphenyl)-2,3-dihydro-1*H*-naphtho[1,2-*e*][1,3]oxazine(Table 2, Entry 4, 4d)**

Brown solid, m.p. 78-79 ^°^C; FT-IR (ATR) ῡ (cm^-1^): 1623, 1596, 1508, 1467, 1229, 1156, 1032, 943, 805 (**Fig. S8**); ^1^H NMR (Acetone-d_6_, 400 MHz) /δ ppm: 7.80-7.82 (m, 2H, Ar-H), 7.70 (d, 1H, ^3^*J*= 8.8 Hz, Ar-H), 7.51 (m, 1H, Ar-H), 7.36 (m, 1H, Ar-H), 7.13 (d, 2H, ^3^*J*= 7.2 Hz, Ar-H), 7.00 (d, 1H, ^3^*J*= 8.8 Hz, Ar-H), 6.80 (d, 2H, ^3^*J*= 7.2 Hz, Ar-H), 5.42 (s, 2H, O-CH_2_-N), 4.93(s, 2H, -Ar-CH_2_-N), 3.68 (s, 3H, O-CH_3_); (**Fig. S9**).

**
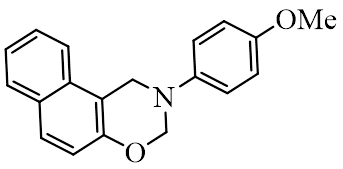
**
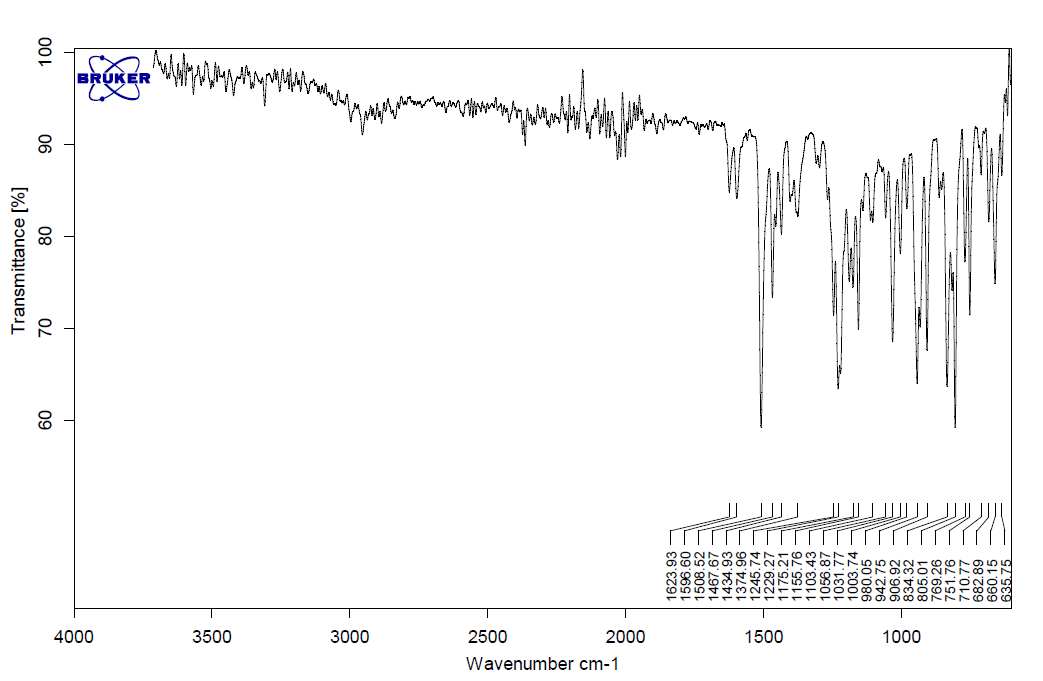


**Fig. S8.** The FT-IR spectrum of product (**4d**)

**
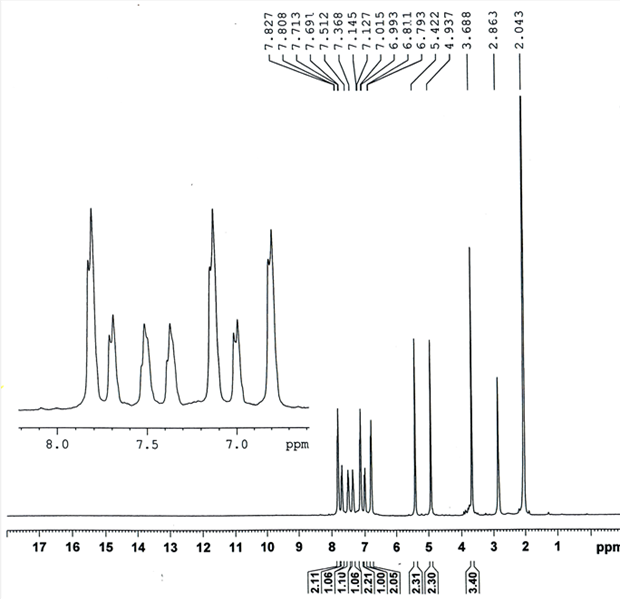
**

**
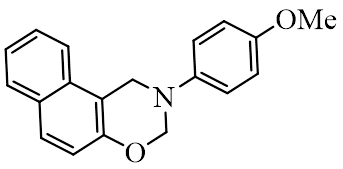
**

**Figure S9**. ^1^H NMR spectrum of compound (**4d**)

**2,3-Dihydro-1*H*-naphtho[1,2-*e*][1,3]oxazine (Table 2, Entry 5, 4e)**

Bold cream solid, m.p. 84-85 ^°^C; FT-IR (ATR) ῡ (cm^-1^): 1625, 1599, 1513, 1227, 936, 807 (**Fig. S10**); ^1^H NMR (Acetone-d_6_, 400 MHz) /δ ppm: 7.82 (t, 2H, ^3^*J*= 8.8 Hz, Ar-H), 7.69 (d, 1H, ^3^*J*= 9.2 Hz, Ar-H), 7.51 (t, 1H, ^3^*J*= 8 Hz, Ar-H), 7.36 (t, 1H, ^3^*J*= 8 Hz, Ar-H), 6.99-7.04 (m, 3H, Ar-H), 7.09-7.11 (m, 2H, Ar-H), 5.52 (s, 2H, O-CH_2_-N), 5.02 (s, 2H, -Ar-CH_2_-N), 2.24 (s, 3H, CH_3_-Ar), (**Fig. S11**).


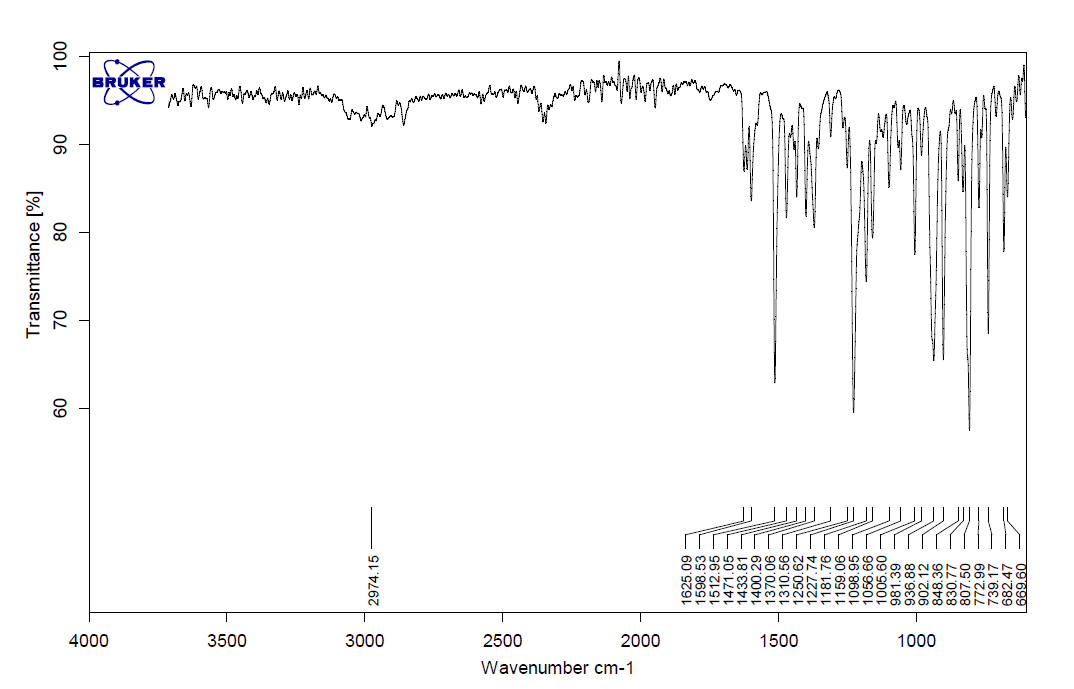


**Fig. S10.** The FT-IR spectrum of product (**4e**)

**Figure S11**. ^1^H NMR spectrum of compound (**4e**)

**2-Cyclohexyl-2,3-dihydro-1*H*-naphtho[1,2-*e*][1,3]oxazine (Table 2, Entry 6, 4f)**

Bold brown solid, m.p. 254 ^°^C (d); FT-IR (ATR) ῡ (cm^-1^): 2927, 2852, 1624, 1599, 1433, 1263, 1058, 862 (**Fig. S12**); ^1^H NMR (DMSO-d_6_, 500 MHz) /δ ppm: 7.66-7.81 (m, 3H, Ar-H), 7.48 (m, 1H, Ar-H), 7.35 (m, 1H, Ar-H), 6.98 (m, 1H, Ar-H), 4.99 (s, 2H, O-CH_2_-N), 4.33 (s, 2H, -Ar-CH_2_-N), 2.70 (m, 1H, CH-N), 1.08-1.86 (m, 10H, 5CH_2_); (**Fig. S13**).

**
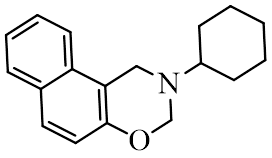
**
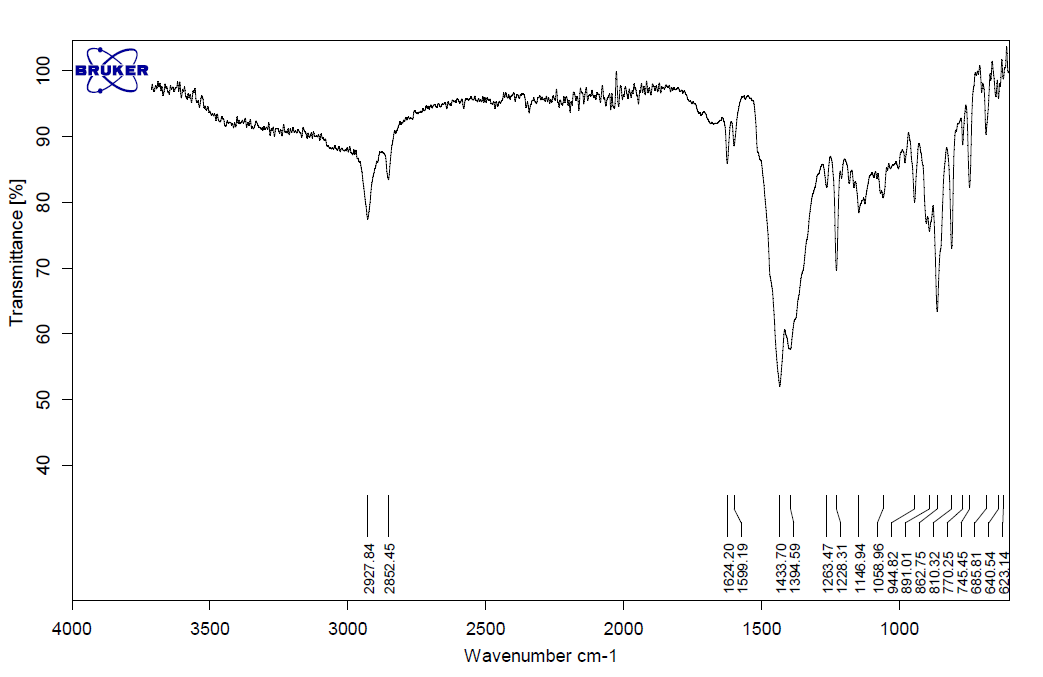


**Fig. S12.** The FT-IR spectrum of product (**4f**)

**
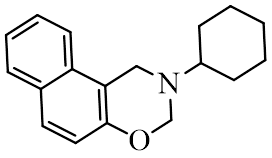

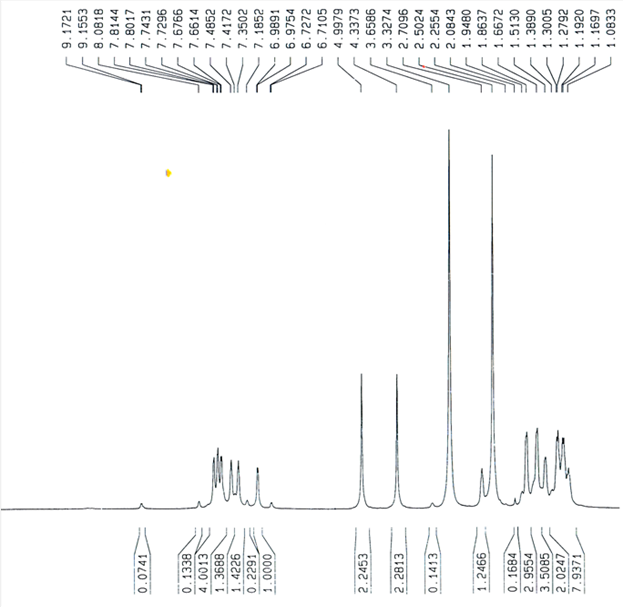
**

**Figure S13**. ^1^H NMR spectrum of compound (**4f**)

**2-Butyl-2,3-dihydro-1*H*-naphtho[1,2-*e*][1,3]oxazine(Table 2, Entry 7, 4g)**

White solid, m.p. 170 ^°^C; FT-IR (ATR) ῡ (cm^-1^): 2955, 2861, 1624, 1598, 1468, 1226, 1057 (**Fig. S14**);

^1^H NMR (DMSO-d_6_, 500 MHz) /δ ppm: 7.81 (m, 1H, Ar-H), 7.69 (m, 2H, Ar-H), 7.47 (m, 1H, Ar-H), 7.35 (m, 1H, Ar-H), 7.00 (m, 1H, Ar-H), 4.88 (s, 2H, O-CH_2_-N), 4.25 (s, 2H, -Ar-CH_2_-N), 2.69 (m, 2H, -CH_2_-N), 1.53 (m, 2H, CH_2_), 1.31 (m, 2H, CH_2_), 0.87 (m, 3H, CH_3_); (**Fig. S15**).

^13^C NMR (DMSO-d_6_, 100 MHz) /δ ppm: 13.80, 19.80, 29.64, 46.94, 50.84, 81.81, 112.08, 118.25, 121.31, 123.26, 126.45, 127.57, 128.32, 128.37, 131.54, 151.51 (**Fig. S16**).

**
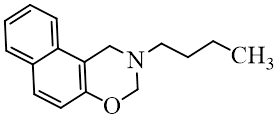
**
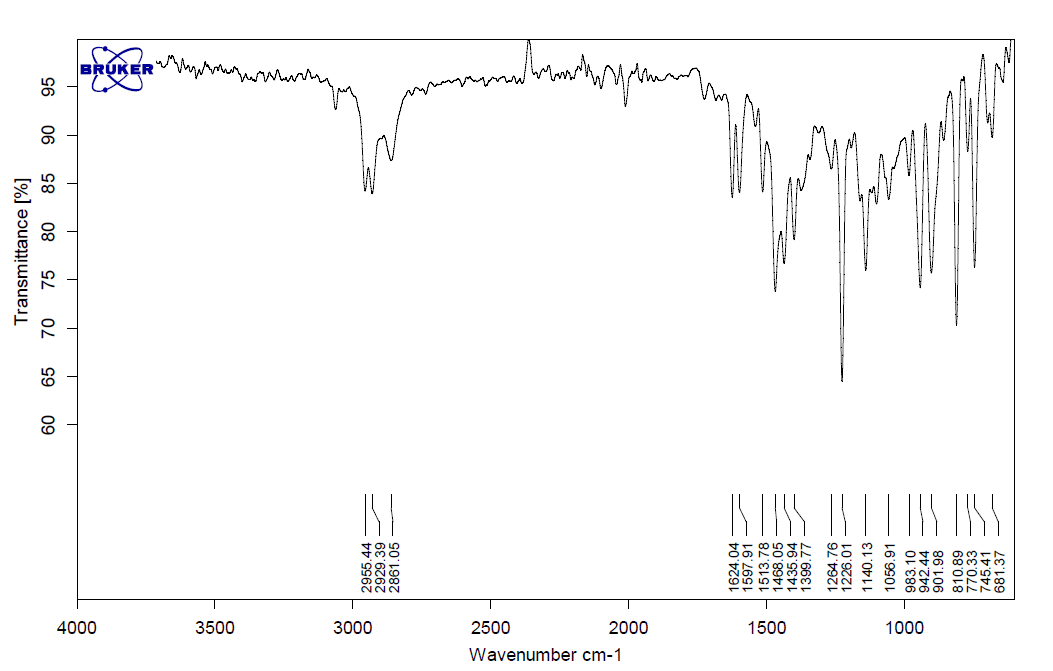


**Fig. S14.** The FT-IR spectrum of product (**4g**)

**
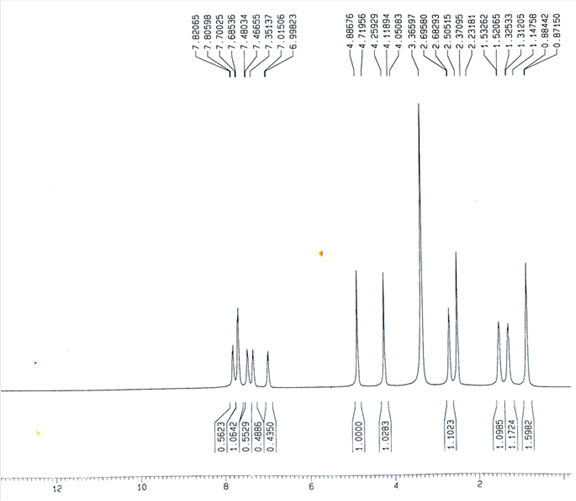
**

**
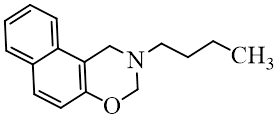
**

**Figure S15**. ^1^H NMR spectrum of compound (**4g**)


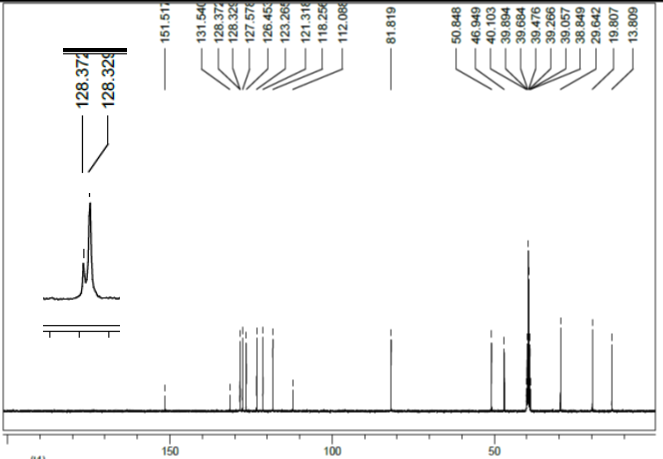


**
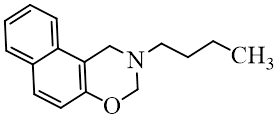
**

**Figure S16**. ^13^C NMR spectrum of compound (**4g**)

**2-Hexyl-2,3-dihydro-1*H*-naphtho[1,2-*e*][1,3]oxazine (Table 2, Entry 8, 4h)**

Cream solid, m.p. 182-183 ^°^C; FT-IR (ATR) ῡ (cm^-1^): 2927, 2855,1625, 1598, 1468, 1226, 1058 (**Fig. S17**); ^1^H NMR (DMSO-d_6_, 500 MHz) /δ ppm: 7.80 (m, 1H, Ar-H), 7.68 (m, 2H, Ar-H), 7.47 (m, 1H, Ar-H), 7.34 (m, 1H, Ar-H), 7.00 (m, 1H, Ar-H), 4.87 (s, 2H, O-CH_2_-N), 4.25 (s, 2H, -Ar-CH_2_-N), 2.68 (m, 2H, -CH_2_-N), 1.53 (m, 2H, CH_2_), 1.25 (m, 6H, 3CH_2_), 0.84 (m, 3H, CH_3_); (**Fig. S18**).

^13^C NMR (DMSO-d_6_, 125 MHz) /δ ppm: 14.77, 22.98, 27.23, 28.38, 32.02, 47.87, 52.09, 82.71, 112.97, 119.14, 122.18, 124.13, 127.32, 128.46, 129.21, 129.28, 132.44, 152.42 (**Fig. S19**).

**
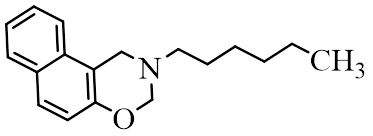
**
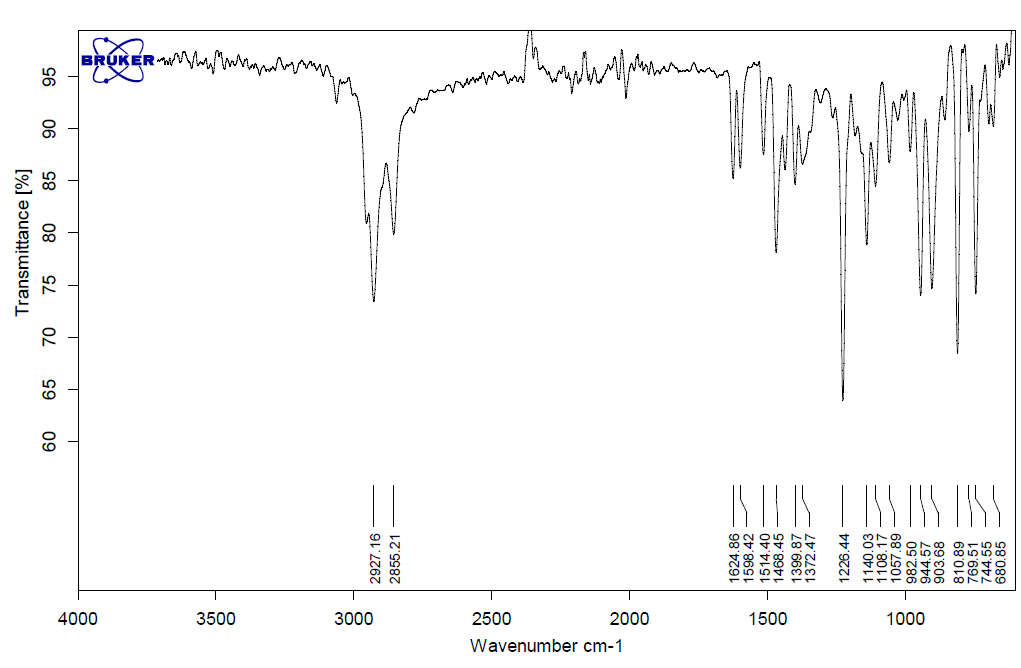


**Fig. S17.** The FT-IR spectrum of product (**4h**)

**
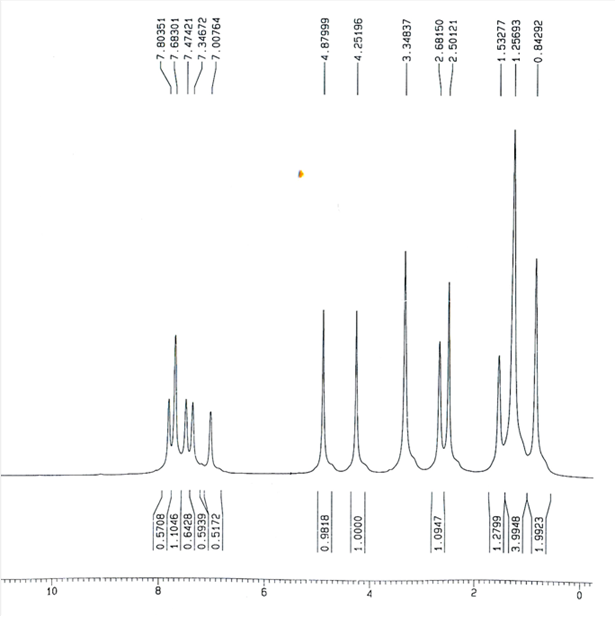

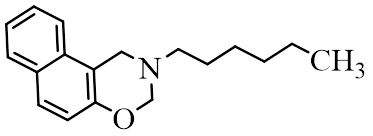
**

**Figure S18**. ^1^H NMR spectrum of compound (**4h**)

**
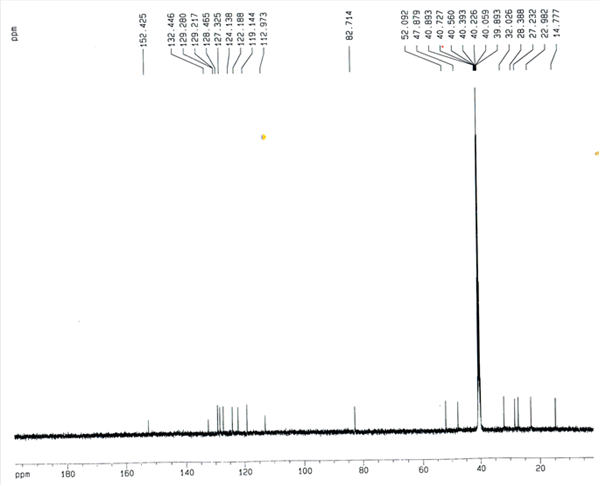
**

**
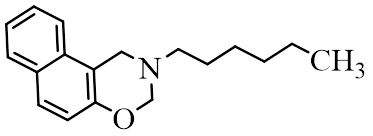
**

**Figure S19**. ^13^C NMR spectrum of compound (**4h**)

**2-(5-Chloro-2-methylphenyl)-2,3-dihydro-1*H*-naphtho[1,2-*e*][1,3]oxazine(Table 2, Entry 9, 4i)**

White solid, m.p. 151-152 ^°^C; FT-IR (ATR) ῡ (cm^-1^): 2918, 1623, 1591, 1470, 1266, 1058 (**Fig. S20**); ^1^H NMR (DMSO-d_6_, 400 MHz) /δ ppm: 7.87 (m, 1H, Ar-H), 7.79 (m, 2H, Ar-H), 7.52 (m, 1H, Ar-H), 7.41(m, 1H, Ar-H), 7.12 (m, 3H, Ar-H), 7.07 (m, 1H, Ar-H), 5.28 (s, 2H, O-CH_2_-N), 4.76 (s, 2H, -Ar-CH_2_-N), 2.5 (m, 2H, -CH_2_-N), 2.40 (m, 2H, CH_2_); (**Fig. S21**).


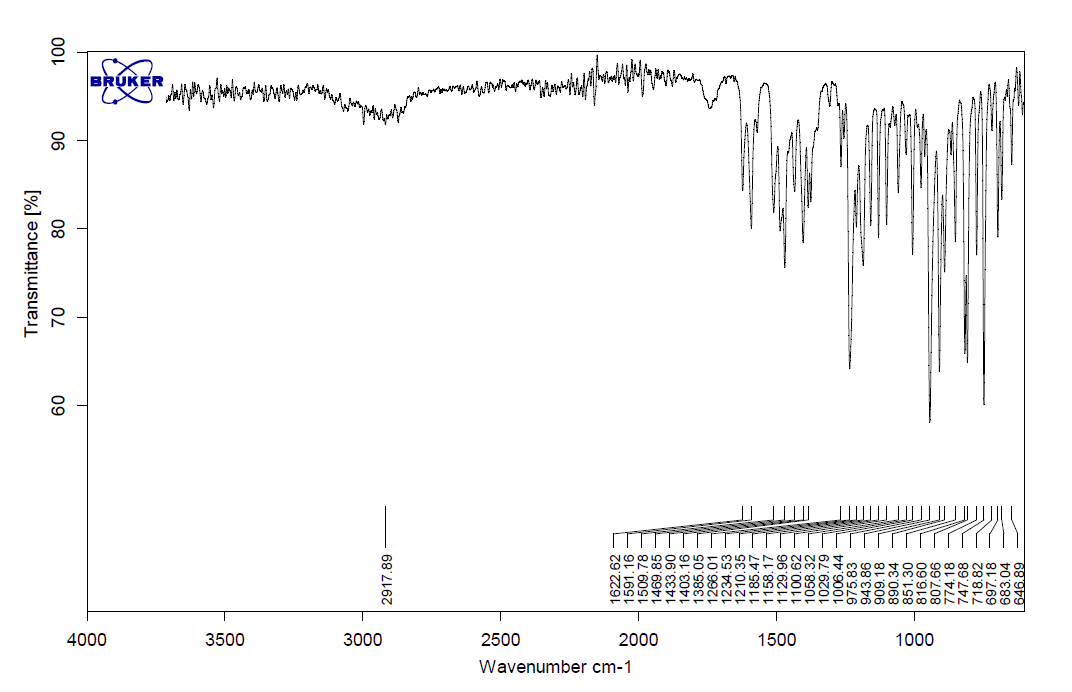


**Fig. S20.** The FT-IR spectrum of product (**4i**)


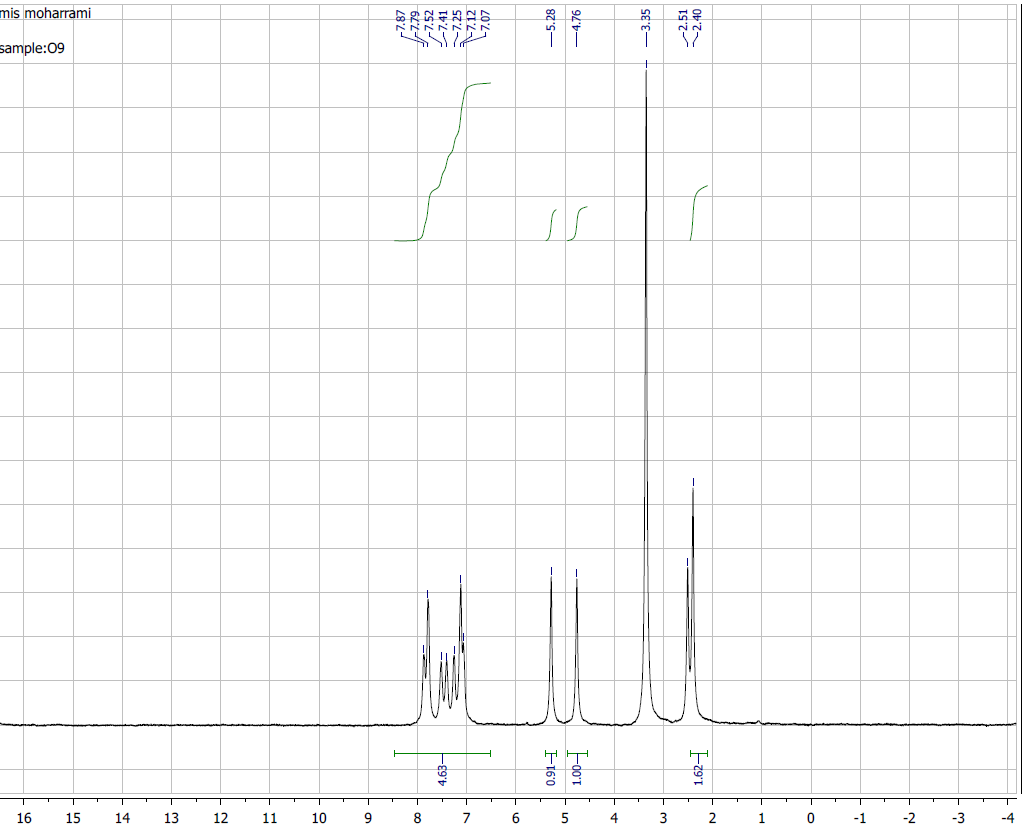


**Figure S21**. ^1^H NMR spectrum of compound (**4i**)

**2-(4-Ethylphenyl)-2,3-dihydro-1*H*-naphtho[1,2-*e*][1,3]oxazine(Table 2, Entry 10, 4j)**

Brown solid, m.p. 48-49 ^°^C; FT-IR (ATR) ῡ (cm^-1^): 2959, 1597, 1511, 1436, 1224, 1060 (**Fig. S22**); ^1^H NMR (Acetone-d_6_, 400 MHz) /δ ppm: 7.80-7.85 (m, 2H, Ar-H), 7.69-7.71 (m, 1H, Ar-H), 7.50-7.55 (m, 1H, Ar-H), 7.35-7.40 (m, 1H, Ar-H), 6.99-7.14 (m, 5H, Ar-H), 5.49 (s, 2H, O-CH_2_-N), 5.00 (s, 2H, -Ar-CH_2_-N), 2.49-2.54 (m, 2H, -CH_2_-CH_3_), 1.15 (t, 3H, ^3^*J*= 7 Hz, -CH_2_-CH_3_); (**Fig. S23**).


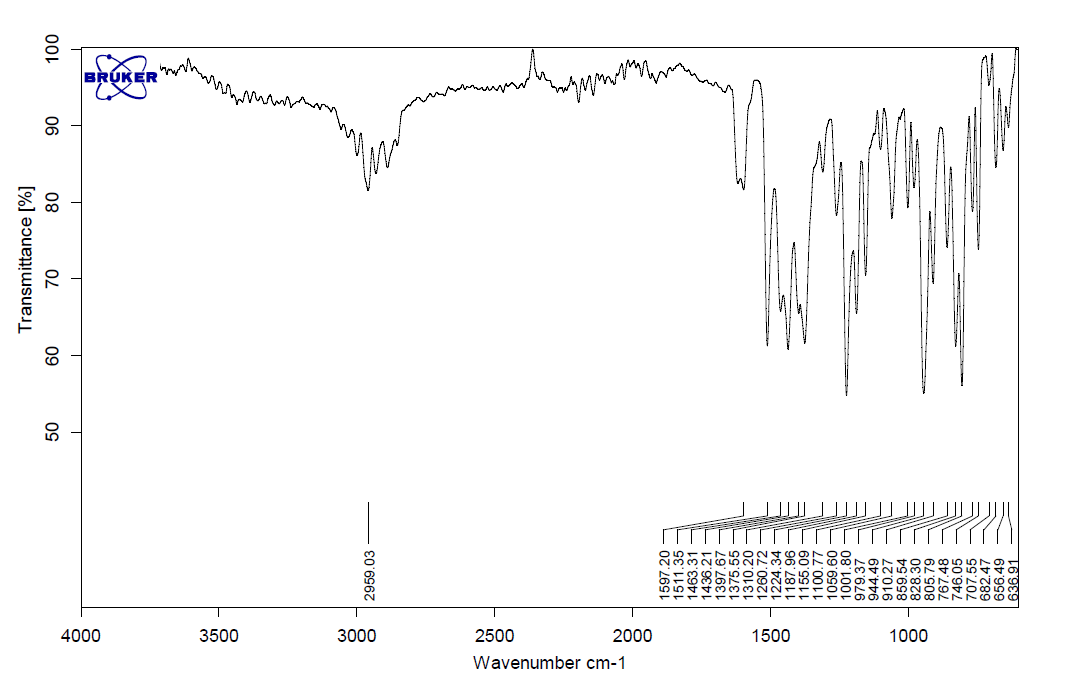


**Fig. S22.** The FT-IR spectrum of product (**4j**)

**Figure S23**. ^1^H NMR spectrum of compound (**4j**)

**2-(2-Chlorobenzyl)-2,3-dihydro-1*H*-naphtho[1,2-*e*][1,3]oxazine(Table 2, Entry 11, 4k)**

Brown solid, m.p. 72-73 ^°^C; FT-IR (ATR) ῡ (cm^-1^): 1621, 1596, 1429, 1227, 1057 (**Fig. S24**); ^1^H NMR (DMSO-d_6_, 500 MHz) /δ ppm: 7.07-7.82 (m, 10H, Ar-H), 4.95 (s, 2H, O-CH_2_-N), 4.27 (s, 2H, -Ar-CH_2_-N), 4.00 (s, 2H, -Ar-CH_2_-N); (**Fig. S25**). ^13^C NMR (DMSO-d_6_, 100 MHz) /δ ppm: 46.82, 52.56, 81.94, 111.73, 118.33, 121.26, 123.42, 126.46, 127.23, 127.85, 128.39, 128.50, 128.90, 129.35, 130.61, 131.54, 133.23, 135.81, 151.41 (**Fig. S26**).

**
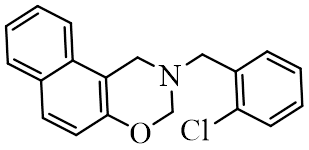
**
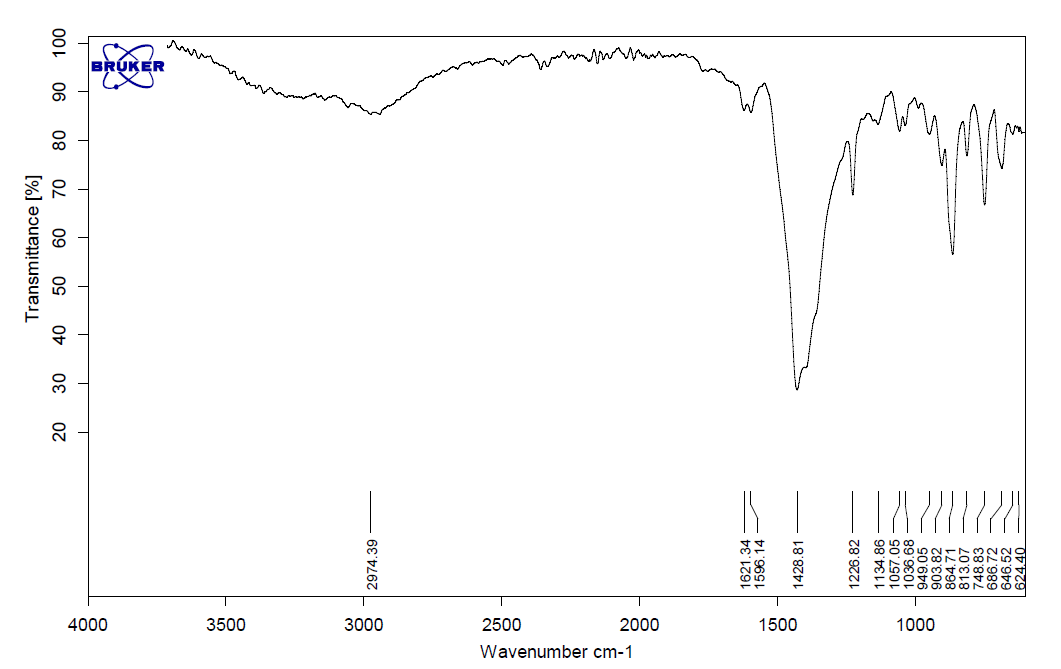


**Fig. S24.** The FT-IR spectrum of product (**4k**)


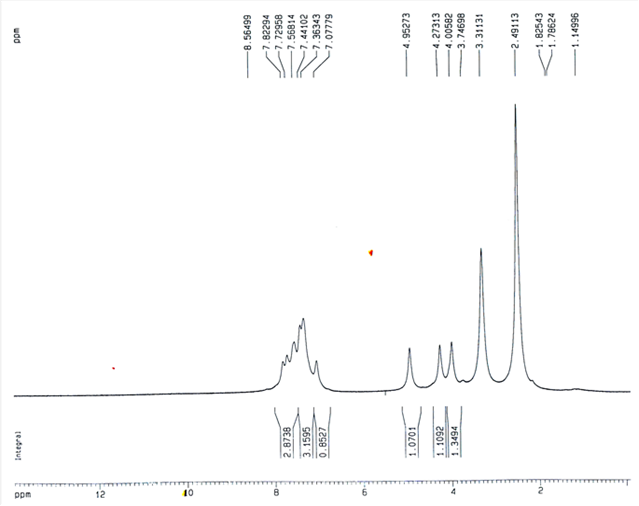


**
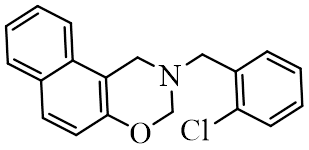
**

**Figure S25**. ^1^H NMR spectrum of compound (**4k**)

**
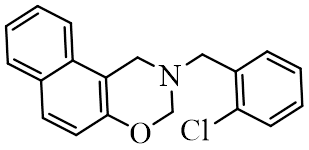

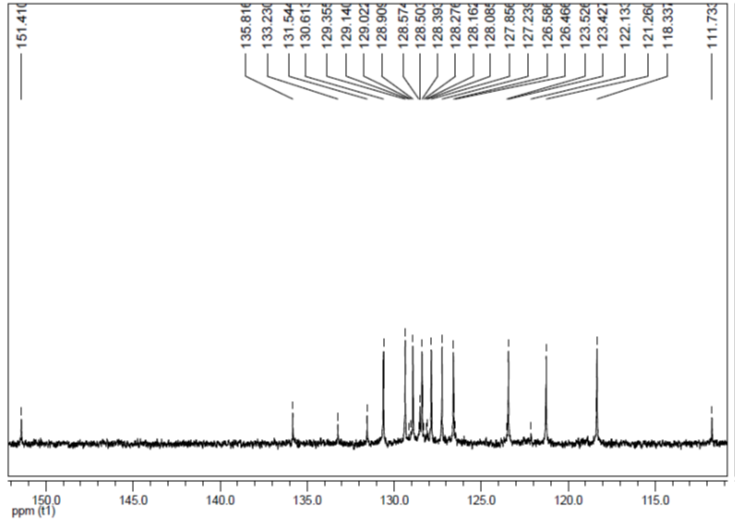
**

**Figure S26**. ^13^C NMR spectrum of compound (**4k**)

**2-Benzyl-2,3-dihydro-1*H*-naphtho[1,2-*e*][1,3]oxazine(Table 2, Entry 12, 4l)**

White solid, m.p. 143-144 ^°^C; FT-IR (ATR) ῡ (cm^-1^): 2943, 1623, 1596, 1462, 1219, 1057, 738 (**Fig. S27**);

^1^H NMR (DMSO-d_6_, 500 MHz) /δ ppm: 7.05-7.71 (m, 11H, Ar-H), 4.89 (s, 2H, O-CH_2_-N), 4.22 (s, 2H, -Ar-CH_2_-N), 3.89 (s, 2H, -Ar-CH_2_-N); (**Fig. S28**). ^13^C NMR (DMSO-d_6_, 100 MHz) /δ ppm: 46.55, 55.17, 81.55, 111.67, 118.32, 121.15, 123.37, 126.57, 127.19, 127.76, 128.34, 128.39, 128.49, 128.59, 131.53, 138.33, 151.41 (**Fig. S29-S30**).


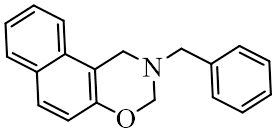

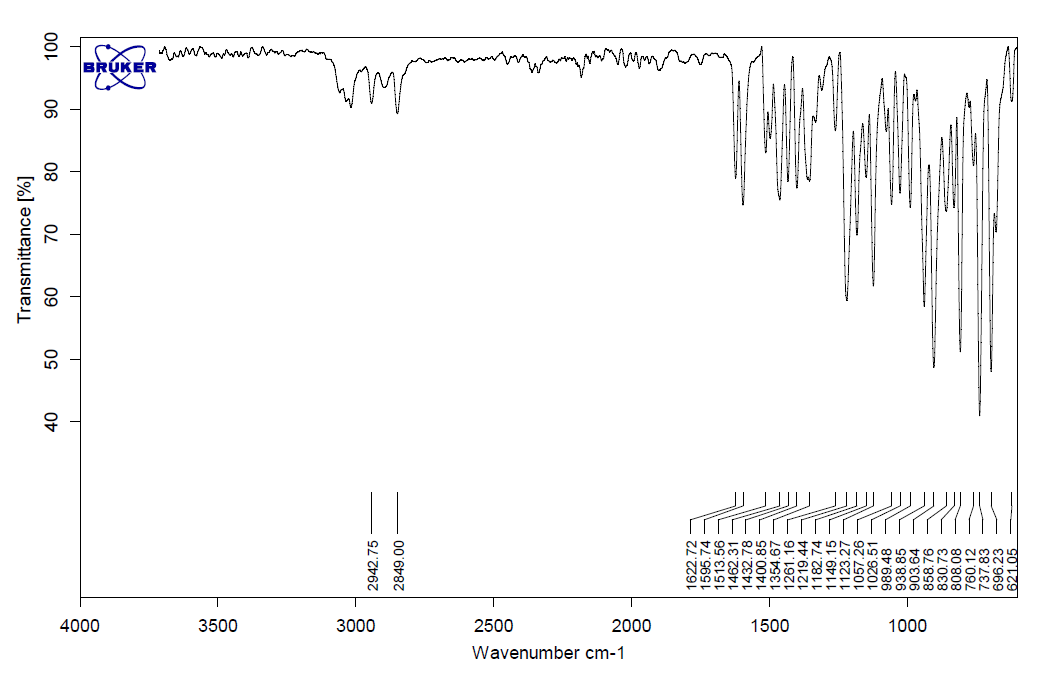


**Fig. S27.** The FT-IR spectrum of product (**4l**)


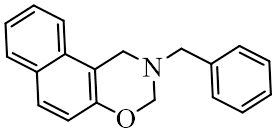

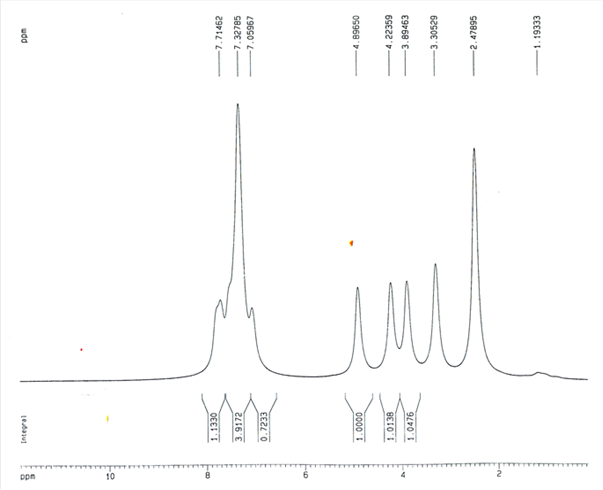


**Figure S28**. ^1^H NMR spectrum of compound (**4l**)


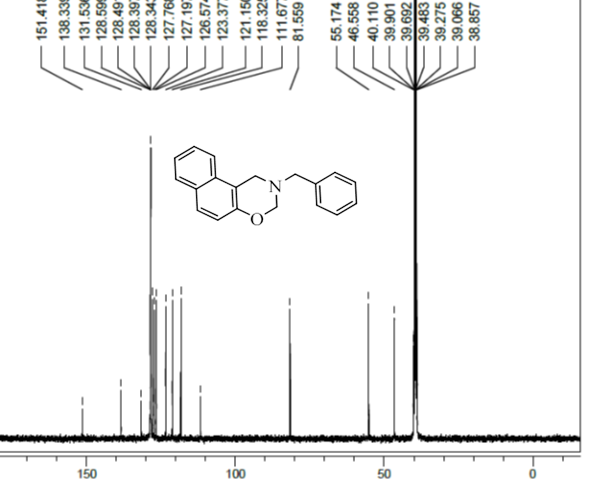


**Figure S29**. ^13^C NMR spectrum of compound (**4l**)


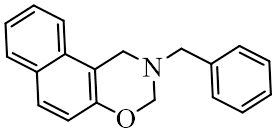

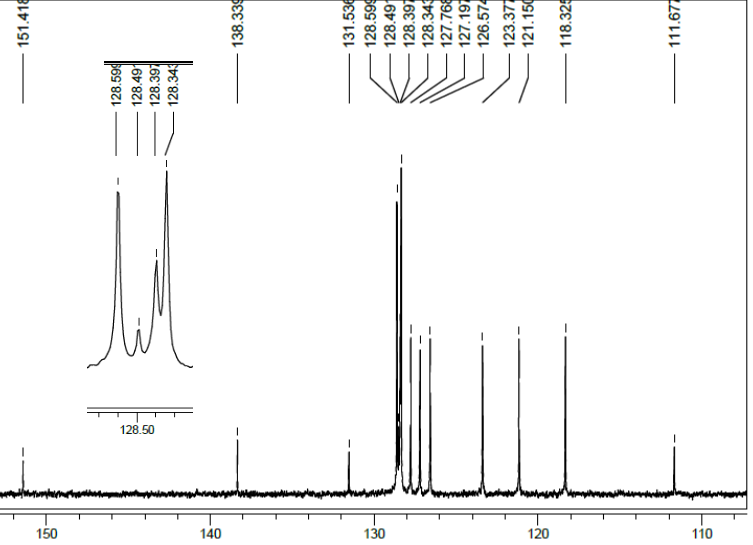


**Figure S30**. ^13^C NMR spectrum of compound (**4l**)

**2-(2-Bromobenzyl)-2,3-dihydro-1*H*-naphtho[1,2-*e*][1,3]oxazine (Table 2, Entry 13, 4m)**

Brown solid, m.p. 120-122 ^°^C; FT-IR (ATR) ῡ (cm^-1^): 2880,1623, 1597, 1507, 1476, 1229,1070 ( **Fig.S31**); ^1^H NMR (DMSO-d_6_, 400 MHz) /δ ppm: 7.91 (t, 2H, ^3^*J*= 9.2 Hz, Ar-H), 7.72 (d, 1H, ^3^*J*= 9.2 Hz, Ar-H), 7.59(t, 1H, ^3^*J*= 8.4 Hz, Ar-H), 7.42 (m, 3H, Ar-H), 7.24(d, 2H, ^3^*J*= 9.2 Hz, Ar-H), 7.03(d, 1H, ^3^*J*= 9.2 Hz, Ar-H), 5.36 (s, 2H, O-CH_2_-N), 4.86 (s, 2H, -Ar-CH_2_-N); (**Fig. S32**).


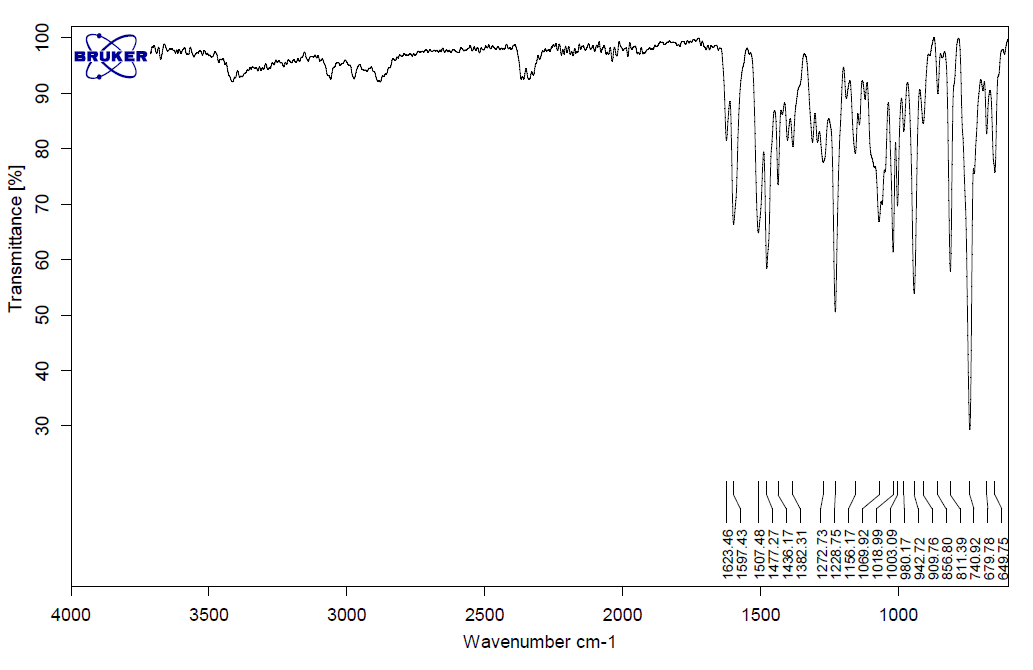


**Fig. S31.** The FT-IR spectrum of product (**4m**)


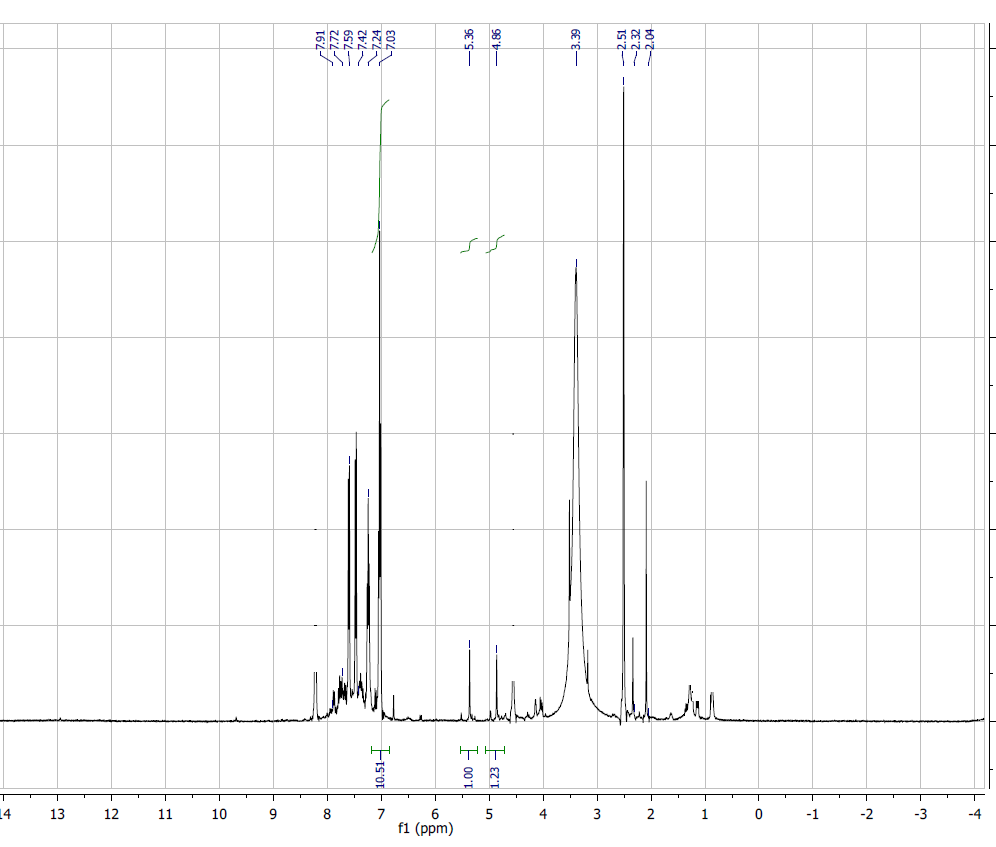


**Figure S32**. ^1^H NMR spectrum of compound (**4m**)
